# Ubiquinone Biosynthesis over the Entire O_2_ Range: Characterization of a Conserved O_2_-Independent Pathway

**DOI:** 10.1128/mBio.01319-19

**Published:** 2019-07-09

**Authors:** Ludovic Pelosi, Chau-Duy-Tam Vo, Sophie Saphia Abby, Laurent Loiseau, Bérengère Rascalou, Mahmoud Hajj Chehade, Bruno Faivre, Mathieu Goussé, Clothilde Chenal, Nadia Touati, Laurent Binet, David Cornu, Cameron David Fyfe, Marc Fontecave, Frédéric Barras, Murielle Lombard, Fabien Pierrel

**Affiliations:** a CNRS, CHU Grenoble Alpes, Grenoble INP, TIMC-IMAG, Université Grenoble Alpes, Grenoble, France; b Laboratoire de Chimie des Processus Biologiques, Collège de France, CNRS UMR 8229, PSL Research University, Sorbonne Université, Paris, France; c CNRS, Laboratoire Chimie Bactérienne, Institut Microbiologie de la Méditerranée, Aix Marseille Université, Marseille, France; d ENSCP-Chimie ParisTech, Institut de Recherche de Chimie Paris, CNRS UMR 8247, Paris, France; e Plateforme SICaPS, Institut de Biologie Intégrative de la Cellule (I2BC), Gif-sur-Yvette, France; f SAMe Unit, Department of Microbiology, Institut Pasteur, Paris, France; California Institute of Technology

**Keywords:** bioenergetics, facultative anaerobes, hydroxylases, iron-sulfur, oxygen, peptidase U32, proteobacteria, quinones, respiration, ubiquinone

## Abstract

Most bacteria can generate ATP by respiratory metabolism, in which electrons are shuttled from reduced substrates to terminal electron acceptors, via quinone molecules like ubiquinone. Dioxygen (O_2_) is the terminal electron acceptor of aerobic respiration and serves as a co-substrate in the biosynthesis of ubiquinone. Here, we characterize a novel, O_2_-independent pathway for the biosynthesis of ubiquinone. This pathway relies on three proteins, UbiT (YhbT), UbiU (YhbU), and UbiV (YhbV). UbiT contains an SCP2 lipid-binding domain and is likely an accessory factor of the biosynthetic pathway, while UbiU and UbiV (UbiU-UbiV) are involved in hydroxylation reactions and represent a novel class of O_2_-independent hydroxylases. We demonstrate that UbiU-UbiV form a heterodimer, wherein each protein binds a 4Fe-4S cluster via conserved cysteines that are essential for activity. The UbiT, -U, and -V proteins are found in alpha-, beta-, and gammaproteobacterial clades, including several human pathogens, supporting the widespread distribution of a previously unrecognized capacity to synthesize ubiquinone in the absence of O_2_. Together, the O_2_-dependent and O_2_-independent ubiquinone biosynthesis pathways contribute to optimizing bacterial metabolism over the entire O_2_ range.

## Abstract (precis)

In order to colonize environments with large O_2_ gradients or fluctuating O_2_ levels, bacteria have developed metabolic responses that remain incompletely understood. Such adaptations have been recently linked to antibiotic resistance, virulence, and the capacity to develop in complex ecosystems like the microbiota. Here, we identify a novel pathway for the biosynthesis of ubiquinone, a molecule with a key role in cellular bioenergetics. We link three uncharacterized genes of Escherichia coli to this pathway and show that the pathway functions independently from O_2_. In contrast, the long-described pathway for ubiquinone biosynthesis requires O_2_ as a substrate. In fact, we find that many proteobacteria are equipped with the O_2_-dependent and O_2_-independent pathways, supporting that they are able to synthesize ubiquinone over the entire O_2_ range. Overall, we propose that the novel O_2_-independent pathway is part of the metabolic plasticity developed by proteobacteria to face various environmental O_2_ levels.

## INTRODUCTION

Since the oxygenation of the Earth’s atmosphere some 2.3 billion years ago, many organisms adopted dioxygen (O_2_) as a terminal electron acceptor of their energy-producing respiratory chains (1). Indeed, oxygenic (aerobic) respiration has an energetic output superior to that of anaerobic respiration or fermentation, which are both O_2_-independent processes (1). In fact, several microorganisms, including many important human pathogens, are facultative anaerobes that are able to adopt either an aerobic or an anaerobic lifestyle depending on the environmental conditions (2, 3). In the laboratory, bacteria are usually cultured and studied under fully aerobic or completely anaerobic conditions (absence of O_2_) (4), whereas natural habitats cover the entire range of O_2_ concentrations (5). For instance, large O_2_ gradients are typically encountered in the human large intestine, in biofilms, or in transition zones between oxic and anoxic environments (5). Moreover, bacteria can experience rapid transitions between environments with vastly different O_2_ contents, such as during the infection process of enteric pathogens that progress along the gastrointestinal tract (3).

To maximize their bioenergetic capacities according to the various levels of O_2_ encountered in their environment, bacteria modulate the composition of their respiratory chains, notably the quinone species and the terminal reductases (3, 4, 6). Quinones are lipophilic redox molecules that fuel electrons to terminal reductases, which reduce O_2_ whenever available, or alternative electron acceptors, for instance, nitrate, dimethyl sulfoxide (DMSO), and trimethylamine N-oxide (7). Naphtoquinones (menaquinone [MK] and demethylmenaquinone [DMK]) and ubiquinone (UQ) are the two main groups of bacterial quinones. (D)MK and UQ differ by the nature of their head group and the value of their redox midpoint potential (8). (D)MK are considered anaerobic quinones, since they function primarily in anaerobic respiration, whereas UQ is considered an aerobic quinone, because it supplies electrons mostly to the reductases that reduce O_2_ (1, 8, 9). Accordingly, UQ is the main quinone of the facultative anaerobe Escherichia coli under aerobic conditions, whereas the naphtoquinones are predominant in the absence of O_2_ (10, 11), with UQ nevertheless being present.

**FIG 1:**
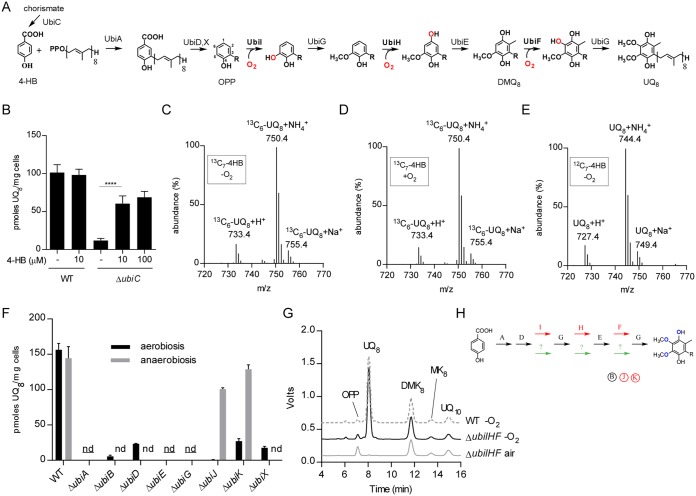
Aerobic and anaerobic UQ biosynthetic pathways differ only in the hydroxylation steps. (A) O_2_-dependent UQ biosynthesis pathway in E. coli. The octaprenyl tail is represented by R on the biosynthetic intermediates, and the numbering of the aromatic carbon atoms is shown on OPP. Abbreviations used are 4-HB for 4-hydroxybenzoic acid, OPP for octaprenylphenol, DMQ_8_ for C6-demethoxy-ubiquinone 8, and UQ_8_ for ubiquinone 8. (B) UQ_8_ quantification of WT and Δ*ubiC* cells grown anaerobically in glycerol-nitrate medium supplemented with the indicated concentrations of 4-HB or left unsupplemented. Values are means ± standard deviations (SD) (*n* = 3 to 6). ****, *P* < 0.0001 by unpaired Student's *t* test. (C to E) Mass spectra of UQ_8_ obtained by HPLC-MS analysis of lipid extracts from cells grown with ^13^C_7_-4-HB either anaerobically (C) or aerobically (D) or anaerobically with unlabeled 4-HB (E). (F) UQ_8_ quantification from WT and Δ*ubi* cells grown anaerobically in SMGN medium overnight or aerobically in LB medium until an OD of 0.8 was reached. nd, not detected under aerobic and anaerobic conditions; nd, not detected under anaerobic conditions. Values are means ± SD (*n* = 3 to 4). (G) HPLC-ECD analyses (mobile phase 1) of lipid extracts from 1 mg of WT or Δ*ubiIHF* cells grown in LB medium under air or anaerobic conditions (−O_2_). Chromatograms are representative of *n* = 3 independent experiments (UQ_10_ used as a standard). (H) UQ biosynthesis represented with Ubi enzymes specific to the O_2_-dependent pathway (red), to the O_2_-independent pathway (green), or common to both pathways (black). The same color code applies to the accessory factors (circled).

The biosynthesis of UQ requires a total of eight reactions to modify the aromatic ring of the precursor, 4-hydroxybenzoic acid (4-HB): one prenylation, one decarboxylation, three hydroxylation, and three methylation reactions (Fig. 1A) (12). In addition to the enzymes that catalyze the various steps, three accessory factors, UbiB, UbiJ, and UbiK, are also needed. UbiB has an ATPase activity (13), and we showed that UbiJ and UbiK (14, 15) belong to a multiprotein UQ biosynthesis complex, in which the SCP2 domain (sterol carrier protein 2) of UbiJ binds the hydrophobic UQ biosynthetic intermediates (16). This UQ biosynthetic pathway is dependent upon O_2_, since all three hydroxylases, UbiI, UbiH, and UbiF, use O_2_ as a cosubstrate (Fig. 1A) (17, 18). We showed recently that other hydroxylases, UbiL, UbiM, and Coq7, replace UbiI, UbiH, and UbiF in some proteobacteria (19). The six hydroxylases have in common their dependence on O_2_ and, thus, function in UQ biosynthesis only when sufficient O_2_ is available. Interestingly, Alexander and Young established 40 years ago that E. coli was able to synthesize UQ anaerobically (20), suggesting the existence of an O_2_-independent biosynthesis pathway, which is still uncharacterized.

In this study, we describe the O_2_-independent UQ biosynthetic pathway in E. coli and identify three essential components, the UbiT, UbiU, and UbiV proteins, formerly called YhbT, YhbU, and YhbV. We show that the O_2_-independent UQ biosynthetic pathway is widely conserved in proteobacteria. UbiT likely functions as an accessory factor in the O_2_-independent UQ biosynthetic pathway, and we show that UbiU and UbiV are involved in at least one O_2_-independent hydroxylation reaction. Moreover, we demonstrate that both UbiU and UbiV bind a [4Fe-4S] cluster essential for activity, which identifies these proteins as prototypes of a new class of O_2_-independent hydroxylases. Our results highlight that many proteobacterial species use two different and complementary molecular pathways to produce UQ over the entire continuum of environmental O_2_.

## RESULTS

### 4-HB is the precursor of UQ synthesized under anaerobic conditions.

4-HB is the precursor of UQ synthesized under aerobic conditions (21). Accordingly, an E. coli Δ*ubiC* mutant impaired in 4-HB biosynthesis (22) is deficient in UQ_8_ and is complemented by addition of 4-HB to the growth medium (23). In order to evaluate whether 4-HB is also the precursor of the O_2_-independent UQ biosynthetic pathway, we grew a Δ*ubiC* strain anaerobically. The Δ*ubiC* strain showed a diminished level of UQ_8_, which was partially recovered by supplementation with 4-HB (Fig. 1B). Furthermore, we grew the Δ*ubiC* strain in medium supplemented with ^13^C_7_-4-HB and analyzed the labeling of biosynthesized UQ_8_ by high-performance liquid chromatography-mass spectrometry (HPLC-MS). In cells grown under aerobic or anaerobic conditions, the labeled form of UQ represented 98.3% and 97.3% (±0.2%), respectively, of the total UQ_8_ pool (Fig. 1C and D). As expected, Δ*ubiC* cells grown anaerobically with unlabeled 4-HB did not show any ^13^C_6_-UQ_8_ (Fig. 1E). Together, these results establish that 4-HB is the precursor of the O_2_-independent UQ biosynthetic pathway.

### Ubi enzymes, except hydroxylases, are common to the aerobic and anaerobic UQ biosynthesis pathways.

The above-described result suggests that the UQ biosynthetic pathways decorate 4-HB with the same chemical groups irrespective of the presence of environmental O_2_. Thus, we evaluated whether the enzymes of the aerobic pathway are also involved in the O_2_-independent pathway by measuring the UQ_8_ content of knockout (KO) strains grown under aerobic and anaerobic conditions. Deletion of *ubiA*, *ubiE*, or *ubiG* abrogated UQ_8_ biosynthesis under both conditions, whereas Δ*ubiB*, Δ*ubiD*, and Δ*ubiX* strains synthesized a limited amount of UQ_8_ but only under aerobic conditions (Fig. 1F). In contrast, *ubiJ* and *ubiK* had no effect on UQ biosynthesis under anaerobic conditions (Fig. 1F).

Under aerobic conditions, the hydroxylation reactions are catalyzed by the flavin monooxygenases (FMOs) UbiF, UbiH, and UbiI that use dioxygen as a cosubstrate (17, 18, 24) (Fig. 1A). We previously reported that cells deleted for a single O_2_-dependent hydroxylase (Δ*ubiF*, Δ*ubiI*, or Δ*ubiH* cells) were deficient in UQ when cultured in the presence of air but synthesized UQ under anaerobic conditions (17), consistent with the existence of an alternative hydroxylation system in the O_2_-independent pathway (20). Indeed, we confirmed that all three FMOs are dispensable for the O_2_-independent UQ biosynthetic pathway, since a Δ*ubiF* Δ*ubiI* Δ*ubiH* triple mutant was deficient for UQ when grown in air but synthesized wild-type (WT) levels of UQ_8_ under anaerobic conditions (Fig. 1G). Together, our results demonstrate that the O_2_-dependent and O_2_-independent UQ biosynthetic pathways share the enzymes involved in the prenylation (UbiA), decarboxylation (UbiX and UbiD), and methylation (UbiE and UbiG) reactions but differ in their hydroxylases and the accessory factors UbiJ and UbiK (Fig. 1H).

### Identification of three genes required for UQ biosynthesis under anaerobic conditions.

**FIG 2:**
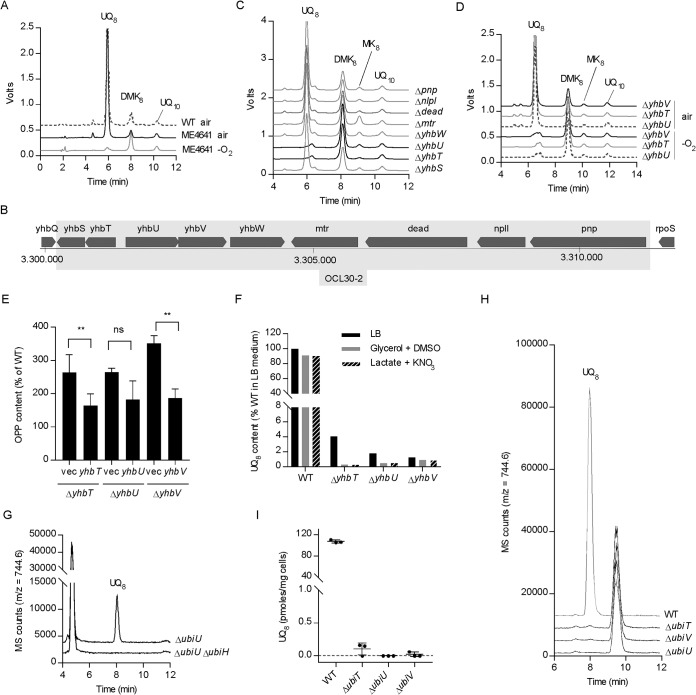
*yhbT*, *yhbU*, and *yhbV* are essential to the anaerobic biosynthesis of UQ. (A) HPLC-ECD analysis of lipid extracts from ME4641 strain grown in SMGN either aerobically or anaerobically (−O_2_). (B) Genomic region covered by the OCL30-2 deletion in the ME4641 strain. (C) HPLC-ECD analysis of lipid extracts from knockout strains of the individual genes covered by the OCL30-2 deletion grown in SMGN anaerobically. (D) HPLC-ECD analysis of lipid extracts from Δ*yhbT*, Δ*yhbU*, and Δ*yhbV* strains constructed in the MG1655 background and grown in SMGN either aerobically or anaerobically. HPLC-ECD analyses with mobile phase 2 (A, C, and D). (E) OPP content (as a percentage of the WT, mass detection M+NH_4_^+^) in cells from Table 1. The Δ*yhb* strains contain either an empty plasmid or a plasmid carrying the indicated gene and were cultured anaerobically in SMGN containing 0.02% arabinose. Values are means ± SD (*n* = 3 to 5). **, *P* < 0.01 by unpaired Student's *t* test. (F) UQ_8_ content (as a percentage of the WT grown in LB medium) of cells cultured anaerobically in SM containing the indicated carbon sources and electron acceptors. (G) Single-ion monitoring for UQ_8_ (M+NH_4_^+^) in HPLC-MS analysis (mobile phase 1) of lipid extracts from 1 mg of Δ*ubiU* or Δ*ubiU* Δ*ubiH* cells grown in SMGN under anaerobic conditions. (H) Single-ion monitoring in HPLC-MS analysis (mobile phase 1) of lipid extracts from 1.6 mg of cells grown in LB medium under strict anaerobic conditions and quenched in methanol. Chromatograms are representative of *n* = 3 independent experiments (G and H). (I) UQ_8_ content of cells described for panel H (quantification of the signal at 8 min with *m/z* 744.6). Values are means ± SD (*n* = 3).

To identify genes involved in the O_2_-independent UQ biosynthetic pathway, we cultivated anaerobically a collection of ∼200 E. coli strains that contained deletions covering multiple open reading frames (ORFs) (25, 26), and we analyzed their UQ_8_ content by HPLC-electrochemical detection (HPLC-ECD). We found a complete absence of UQ_8_ in strains ME4561, ME5034, and ME4746 that carry deletions encompassing *ubiE-ubiJ-ubiB-ubiD*, *ubiG*, and *ubiX*, respectively (see Table S1 in the supplemental material). Several other strains had a low UQ_8_ content and poor growth in synthetic medium supplemented with glycerol and nitrate (SMGN). However, those strains showed better growth and higher UQ_8_ content in LB medium (Table S1). Thus, we did not investigate them further, as a genetic defect affecting directly the O_2_-independent UQ pathway was unlikely. In contrast, ME4641 showed a profound UQ_8_ deficiency and robust anaerobic growth in LB and SMGN media (Table S1). Importantly, ME4641 had a WT UQ_8_ level when grown aerobically, suggesting that only the O_2_-independent pathway was altered (Fig. 2A). ME4641 contains a deletion named OCL30-2 that covers 9 genes, 5 of them lacking an identified function (Fig. 2B). To find the candidate gene involved in the anaerobic biosynthesis of UQ, we obtained 8 single-gene KO strains from the Keio collection (27) and analyzed their quinone content after anaerobic growth (Fig. 2C). The Δ*yhbT* and Δ*yhbU* strains were strongly deficient in UQ_8_. We then transduced the Δ*yhbT* and Δ*yhbU* mutations from the Keio strains into an MG1655 genetic background and also constructed the Δ*yhbV* strain, which was not available in the Keio collection. We found that all three strains had very low levels of UQ_8_ when grown under anaerobic conditions but showed normal levels after aerobic growth (Fig. 2D, Table 1). In addition, the mutant strains showed a 2-fold decrease in MK_8_ and a 2-fold increase in DMK_8_ after anaerobic growth (Table 1). This effect might indirectly result from the UQ_8_ deficiency, as it was also observed in the Δ*ubiG* strain (Fig. S1A).

**TABLE 1:** Quinone content of WT and Δ*yhb* strains cultured in SMGN either aerobically or anaerobically^*a*^

Culture condition	Strain	Quinone content		
		UQ_8_ (pmol/mg cells)	DMK_8_ (247-nm peak area/mg cells)	MK_8_ (247-nm peak area/mg cells)
Air	WT	208 ± 9.4	0.94 ± 0.26	0.15 ± 0.09
Air	Δ*yhbT*	193.9 ± 12.4	1.34 ± 0.05	0.13 ± 0.03
Air	Δ*yhbU*	233.8 ± 16.2	0.9 ± 0.26	0.16 ± 0.09
Air	Δ*yhbV*	227.9 ± 25.1	0.93 ± 0.15	0.13 ± 0.04
Anaerobic	WT	114.2 ± 19.1	1.28 ± 0.4	0.41 ± 0.15
Anaerobic	Δ*yhbT*	3.3 ± 0.4	2.88 ± 0.47	0.17 ± 0.01
Anaerobic	Δ*yhbU*	6.1 ± 3.3	2.55 ± 0.55	0.19 ± 0.01
Anaerobic	Δ*yhbV*	7.3 ± 4.7	2.61 ± 0.49	0.21 ± 0.01
Anaerobic	Δ*yhbT*+pK	2 ± 0.4	2.46 ± 0.33	0.16 ± 0.04
Anaerobic	Δ*yhbT*+pK-yhbT	64.6 ± 16.1	1.77 ± 0.29	0.3 ± 0.04
Anaerobic	Δ*yhbU*+pBAD	1 ± 0.6	2.29 ± 0.58	0.18 ± 0.04
Anaerobic	Δ*yhbU*+pBAD-yhbU	82 ± 15.9	1.2 ± 0.18	0.27 ± 0.04
Anaerobic	Δ*yhbV*+pBAD	2.2 ± 3.5	2.14 ± 0.15	0.23 ± 0.01
Anaerobic	Δ*yhbV*+pBAD-yhbV	120.4 ± 18.5	1.87 ± 0.19	0.42 ± 0.06

a *n* = 3 to 5. For strains containing pK or pBAD vectors, SMGN was supplemented with 0.02% arabinose.

10.1128/mBio.01319-19.1 FIG S1 (A) HPLC-ECD analyses (mobile phase 2) of lipid extracts from 1 mg of WT, Δ*ubiT*, and Δ*ubiG* cells grown in LB medium under anaerobic conditions (UQ_10_ used as the standard). (B) Genetic context of the *ubiT*, *ubiU*, and *ubiV* genes in *M. marinus* (WP_011715033.1). The scheme was drawn from a figure obtained using the GeneSpy program (81). Download FIG S1, JPG file, 0.1 MB. Copyright © 2019 Pelosi et al. 2019 Pelosi et al. This content is distributed under the terms of the Creative Commons Attribution 4.0 International license.

10.1128/mBio.01319-19.7 TABLE S1 UQ levels in strains from the medium- and large-deletion collections. Download Table S1, XLSX file, 0.02 MB. Copyright © 2019 Pelosi et al. 2019 Pelosi et al. This content is distributed under the terms of the Creative Commons Attribution 4.0 International license.

### Deletion of *yhbT*, *yhbU*, or *yhbV* causes UQ_8_ deficiency specifically under anaerobic conditions.

We then transformed the mutant strains with an empty vector or a vector carrying a WT allele of the studied gene. In *yhb* KO strains expressing the corresponding gene from the plasmid, we observed a complementation of the UQ_8_ deficiency (Table 1) and a normalization of the levels of octaprenylphenol (OPP), an early UQ_8_ biosynthetic intermediate (Fig. 2E). The ∼3-fold elevation of OPP in the *yhbT*, *-U*, and *-V* KO mutants suggested that the O_2_-independent UQ biosynthetic pathway was blocked downstream of OPP in these strains. In these experiments, no cross-complementation was observed, for example, the plasmid with *yhbV* had no effect on Δ*yhbT* or Δ*yhbU* strains, suggesting the absence of redundancy in the function of each gene. We also measured a profound UQ_8_ deficiency when the Δ*yhbT*, Δ*yhbU*, and Δ*yhbV* strains were grown anaerobically in various media (glycerol plus DMSO, lactate plus KNO_3_) (Fig. 2F), showing that the UQ_8_ biosynthetic defect is not linked to a particular carbon source or electron acceptor. Altogether, our results demonstrate that the *yhbT*, *yhbU*, and *yhbV* genes are part of the O_2_-independent UQ biosynthetic pathway, so we propose to rename them *ubiT*, *ubiU*, and *ubiV*, respectively.

### Complete absence of UQ biosynthesis in Δ*ubiT*, Δ*ubiU*, and Δ*ubiV* mutants grown under strict anaerobic conditions.

As the Δ*ubiT*, Δ*ubiU*, and Δ*ubiV* strains still contained small amounts of UQ_8_ after growth under anaerobic conditions (Table 1 and Fig. 2F), we wondered how this UQ_8_ was synthesized. To verify if the O_2_-dependent pathway contributed to this synthesis, we inactivated the *ubiH* gene in the Δ*ubiU* strain. Sensitive HPLC-MS detection established that UQ_8_ was completely absent from extracts from the Δ*ubiH* Δ*ubiU* cells (Fig. 2G). This result supported that the residual UQ_8_ synthesized in Δ*ubiU* cells originated from the O_2_-dependent pathway and suggested that our anaerobic media contain trace amounts of O_2_ or that O_2_-dependent UQ biosynthesis occurs during the handling of cells, under normal atmosphere, prior to quinone extraction.

To eliminate the traces of O_2_, we took extra precautions in the degassing and inoculation of our media (see Materials and Methods) and also added a reductant (l-cysteine). In addition, we used the redox indicator resazurin to verify strict anaerobiosis during the entire culture. We also modified our sampling procedure to rapidly quench the anaerobic cells in ice-cold methanol in order to prevent any O_2_-dependent UQ biosynthesis prior to quinone extraction. HPLC-MS analysis of extracts from cells cultivated and handled under such strict anaerobic conditions showed the nearly complete absence of UQ_8_ in Δ*ubiT*, Δ*ubiU*, and Δ*ubiV* strains (Fig. 2H and I). In contrast, the UQ_8_ level of the WT strain (Fig. 2I) was comparable to those we measured previously in WT cells cultivated under suboptimal anaerobic conditions (Table 1) (14), establishing that UQ biosynthesis occurred independently from O_2_. Together, our results show that Δ*ubiT*, Δ*ubiU*, and Δ*ubiV* cells are unable to synthesize UQ under strict anaerobic conditions, unlike WT cells. Furthermore, our results support that the low residual UQ content previously observed in Δ*ubiT*, Δ*ubiU*, and Δ*ubiV* strains (Table 1 and Fig. 2F) resulted from the function of the O_2_-dependent pathway.

### *ubiT*, *-U*, and *-V* strongly cooccur exclusively in genomes with potential for UQ biosynthesis.

We investigated the distribution of *ubiT*, -*U*, and -*V* in a large genome data set of 5,750 genomes of bacteria and archaea. We found no evidence of genomes harboring matches for more than one of the three genes of interest outside of *Alphaproteobacteria*, *Betaproteobacteria*, and *Gammaproteobacteria* and three genomes of *Acidithiobacillia* (Table S2A). Interestingly, the three former classes are the only ones known so far to be able to produce UQ (1, 8), consistent with a specific link between UbiT, -U, and -V and UQ biosynthesis.

10.1128/mBio.01319-19.8 TABLE S2 Occurrence of genes from the UQ pathways in bacterial genomes. Download Table S2, XLSX file, 0.5 MB. Copyright © 2019 Pelosi et al. 2019 Pelosi et al. This content is distributed under the terms of the Creative Commons Attribution 4.0 International license.

**FIG 3:**
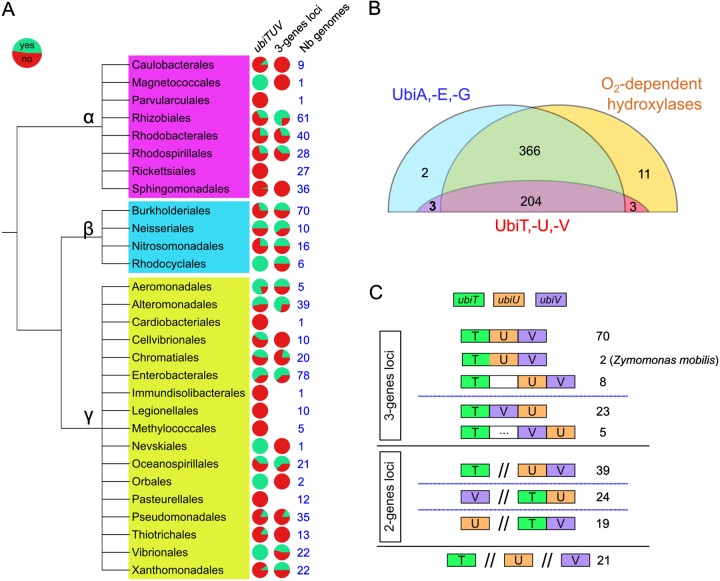
*ubiT*, -*U*, and -*V* occurrence and genetic architecture in proteobacterial genomes. (A) The proportion of genomes with (green) and without (red) all three genes, *ubiTUV* (left column), is indicated for each proteobacterial order known to synthesize UQ. The middle column, labeled 3-genes loci, displays the proportion of genomes with the three genes either at a single locus (green) or at different loci (red). The number of genomes analyzed for each order is given in the right column (Nb genomes). (B) Occurrence in the reference proteobacterial genomes of the marker proteins (UbiA, -E, and -G), of the O_2_-dependent hydroxylases, and of the UbiT,-U, and -V proteins. The number in boldface represents the 3 genomes (*P. fulvum*, *M. marinus*, and *O. formigenes*) containing exclusively the O_2_-independent pathway. (C) The distinct genetic architectures found for *ubiT*, -*U*, and -*V* in genomes where the three genes were present are displayed as boxes with different colors. The numbers of cases corresponding to each depicted architecture are given on the right. A white box corresponds to a gene found between the genes of interest, and a white box with dots corresponds to two to five genes between the genes of interest.

This was confirmed by analyzing in more detail the distribution of *ubiT*, -*U*, and -*V* and three marker genes of the UQ biosynthetic pathway (*ubiA*, *ubiE*, and *ubiG*) in 611 representative and reference genomes of alpha-, beta-, and gammaproteobacteria (Table S2B). We chose *ubiA*, *-G*, and *-E* because they have a higher conservation than other genes, like *ubiC*, *-D*, and *-X* (28), and because they are part of the O_2_-dependent and O_2_-independent pathways (Fig. 1H). A total of 575 genomes had positive matches for *ubiA*, *-G*, and *-E*, and 589 had positive matches for at least two of them. Regarding the distribution of *ubiT*, -*U*, and -*V*, 221 genomes had matches for at least one of the three genes, and in 210 cases (95%) the three genes were present. Importantly, all genomes with *ubiT*, -*U*, and -*V* harbored at least two of the three marker genes for the UQ biosynthetic pathway (Table S2B). In addition, we found that 22 out of the 29 proteobacterial orders analyzed had up to 50% of genomes harboring a complete set of the *ubiT*, -*U*, and -*V* genes (Fig. 3A), demonstrating a wide taxonomic distribution. Overall, our analysis indicates a strong pattern of cooccurrence of *ubiT*, -*U*, and -*V* and demonstrates that they are uniquely found in genomes showing signs of UQ production.

We found *ubiA*, *-G*, and *-E* and O_2_-dependent hydroxylase genes in 570 genomes, of which 204 also contained *ubiT*, -*U*, and -*V* (Table S2B and Fig. 3B). Only 3 species, Phaeospirillum fulvum, Magnetococcus marinus, and Oxalobacter formigenes, seem to rely exclusively on the O_2_-independent pathway for UQ production, as their genomes contain the *ubiT*, -*U*, and -*V* genes but no O_2_-dependent hydroxylases (Fig. 3B). We noticed that the *ubiT*, -*U*, and -*V* genes are found next to other *ubi* genes in *M. marinus* (Fig. S1B). Interestingly, *P. fulvum* and *M. marinus* were described to synthesize UQ under microaerobic conditions (29–31), and *O. formigenes* has been documented as an obligate anaerobe (32). Together, our results show that the O_2_-independent UQ biosynthesis pathway is widespread in alpha-, beta-, and gammaproteobacterial orders and coexists with the O_2_-dependent pathway in 98% of the cases.

We then looked into the relative positioning of *ubiT*, -*U*, and -*V* in the 210 genomes harboring the three genes. We found 106 cases, covering alpha-, beta-, and gammaproteobacterial orders, where they were located next to each other (termed 3-gene loci) and 82 cases of a 2-gene locus, with the third gene being elsewhere in the genome (Fig. 3C). Evaluation of the genetic architecture of *ubiT*, -*U*, and -*V* revealed that *ubiU* and *ubiV* were found exactly next to each other in 69% of the loci (in all 3-gene loci and in 39/82 of the 2-gene loci) and that the three genes were located in three separate parts of the genome in only 21 cases (Fig. 3C). Interestingly, as an additional support for their involvement in the same function, we found an example of a gene fusion between *ubiT* and *ubiU* in two genomes from Zymomonas mobilis strains (alphaproteobacteria), which also contain a *ubiV* gene directly downstream of the fused gene (Fig. 3C).

### UbiT is an SCP2 protein, and UbiU-UbiV are required for the O_2_-independent hydroxylation of DMQ_8_.

We then analyzed the sequences of the UbiT, UbiU, and UbiV proteins. The major part of UbiT (amino acids [aa] 45 to 133, from a total of 174 aa in the E. coli protein) corresponds to an SCP2 domain (Pfam entry PF02036) (Fig. S2). SCP2 domains typically form a hydrophobic cavity that binds various lipids (33), and our sequence alignment indeed showed the conservation of hydrophobic amino acids at several positions in the SCP2 domain of UbiT (Fig. S2). Recently, we reported that UbiJ binds UQ biosynthetic intermediates in its SCP2 domain and organizes a multiprotein complex composed of several Ubi enzymes (16). We propose that UbiT and its SCP2 domain fulfill similar functions in the O_2_-independent UQ pathway, as UbiJ is required exclusively for the O_2_-dependent biosynthesis of UQ (Fig. 1F).

10.1128/mBio.01319-19.2 FIG S2 Multiple-sequence alignment of UbiT from representative proteobacteria. Sequences were aligned using Mafft (linsi), and the output was generated using Jalview and Inkscape. Hydrophobic residues are colored blue. The position of the SCP2 domain (PF02036) is indicated by a red box. Genbank accession numbers: Vibrio cholerae, NP_230303.1; Pseudomonas aeruginosa, NP_252600.1; Escherichia coli, NP_312065.1; Yersinia pestis, YP_002348366.1; Ralstonia solanacearum, WP_011004260.1; Dechloromonas aromatica, WP_011285876.1; Thiobacillus denitrificans, WP_011312695.1; Rhodopseudomonas palustris, WP_011666115.1; Halorhodospira halophila, WP_011813831.1; Rhodobacter sphaeroides, WP_011909347.1; Aeromonas salmonicida, WP_005310396.1; Paracoccus denitrificans, WP_011750456.1; Neisseria sicca, WP_080614297.1; Phaeospirillum fulvum, WP_074767212.1. Download FIG S2, JPG file, 0.8 MB. Copyright © 2019 Pelosi et al. 2019 Pelosi et al. This content is distributed under the terms of the Creative Commons Attribution 4.0 International license.

**FIG 4:**
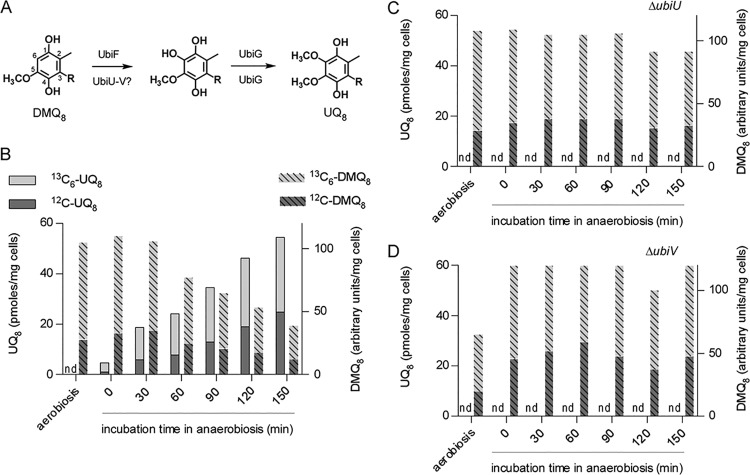
UbiU and UbiV are necessary for the anaerobic conversion of DMQ_8_ into UQ_8_. (A) Conversion of DMQ_8_ to UQ_8_ with enzymes of the O_2_-dependent and the O_2_-independent pathways, indicated above and below arrows (numbering of carbon atoms shown on DMQ_8_ and polyprenyl tail represented by R). (B) Quantification by HPLC-MS (monitoring of Na^+^ adducts) of unlabeled (^12^C) and labeled (^13^C_6_) DMQ_8_ and UQ_8_ in Δ*ubiC* Δ*ubiF* cells after aerobic growth and transition to anaerobiosis. (C and D) Same as panel B but with Δ*ubiC* Δ*ubiF* Δ*ubiU* cells (C) and Δ*ubiC* Δ*ubiF* Δ*ubiV* cells (D). nd, not detected. Results are representative of two independent experiments (B to D).

UbiU and UbiV have ∼330 and 300 aa, respectively, and contain an uncharacterized motif called peptidase U32 (PF01136) (Fig. S3 and S4). Since only the hydroxylation reactions are uncharacterized in the O_2_-independent pathway (Fig. 1H), we hypothesized that UbiU and UbiV function in these steps. To test our hypothesis, we developed an *in vivo* assay based on the O_2_-independent conversion of labeled DMQ_8_ into labeled UQ_8_. This assay monitors the C6-hydroxylation and the subsequent O6-methylation (Fig. 4A). Δ*ubiC* Δ*ubiF* cells grown aerobically with ^13^C_7_-4-HB synthesized DMQ_8_, 73% of which was labeled with ^13^C_6_. Upon transfer to anaerobic conditions, the cells gradually converted a significant part of (^13^C_6_)-DMQ_8_ into (^13^C_6_)-UQ_8_ (Fig. 4B). Inactivation of either *ubiU* or *ubiV* in Δ*ubiC* Δ*ubiF* cells did not perturb the accumulation of (^13^C_6_)-DMQ_8_ but prevented its conversion into (^13^C_6_)-UQ_8_ (Fig. 4C and D). This result demonstrates that UbiU and UbiV are essential for the C6-hydroxylation reaction of the O_2_-independent UQ biosynthetic pathway.

10.1128/mBio.01319-19.3 FIG S3 Multiple-sequence alignment of UbiU from representative proteobacteria. Sequences were aligned using Mafft (linsi), and the output was generated using ESPript and Inkscape. The four conserved cysteines (C169, C176, C193, and C232) involved in iron-sulfur binding are indicated by black columns. The position of the domain U32 protease PF01136 is indicated by a green box. GenBank accession numbers: Vibrio cholerae, NP_230301.1; Pseudomonas aeruginosa, NP_252602.1; Escherichia coli, NP_312066.1; Yersinia pestis, YP_002348368.1; Ralstonia solanacearum, WP_011004262.1; Dechloromonas aromatica, WP_011285878.1; Thiobacillus denitrificans, WP_011312698.1; Rhodopseudomonas palustris, WP_011666114.1; Halorhodospira halophila, WP_011813833.1; Rhodobacter sphaeroides, WP_011909346.1; Aeromonas salmonicida, WP_005310394.1; Paracoccus denitrificans, WP_041530457.1; Neisseria sicca, WP_049226489.1; Phaeospirillum fulvum, WP_074767209.1. Download FIG S3, JPG file, 0.6 MB. Copyright © 2019 Pelosi et al. 2019 Pelosi et al. This content is distributed under the terms of the Creative Commons Attribution 4.0 International license.

10.1128/mBio.01319-19.4 FIG S4 Multiple-sequence alignment of UbiV from representative proteobacteria. Sequences were aligned using Mafft (linsi), and the output was generated using ESPript and Inkscape. The four conserved cysteines (C39, C180, C193, and C197 [positions in E. coli]) involved in iron-sulfur binding are indicated by black columns. The position of the domain U32 protease PF01136 is indicated by a green box. GenBank accession numbers: Vibrio cholerae, NP_230300.2; Pseudomonas aeruginosa, NP_252601.1; Escherichia coli, NP_312067.2; Yersinia pestis, YP_002348369.1; Ralstonia solanacearum, WP_011004261.1; Dechloromonas aromatica, WP_011285877.1; Thiobacillus denitrificans, WP_011312697.1; Rhodopseudomonas palustris, WP_011666113.1; Halorhodospira halophila, WP_011813832.1; Rhodobacter sphaeroides, WP_011909345.1; Aeromonas salmonicida, WP_005310392.1; Paracoccus denitrificans, WP_011750458.1; Neisseria sicca, WP_080614296.1; Phaeospirillum fulvum, WP_074767477.1. Download FIG S4, JPG file, 0.5 MB. Copyright © 2019 Pelosi et al. 2019 Pelosi et al. This content is distributed under the terms of the Creative Commons Attribution 4.0 International license.

### UbiV contains a [4Fe-4S] cluster.

**FIG 5:**
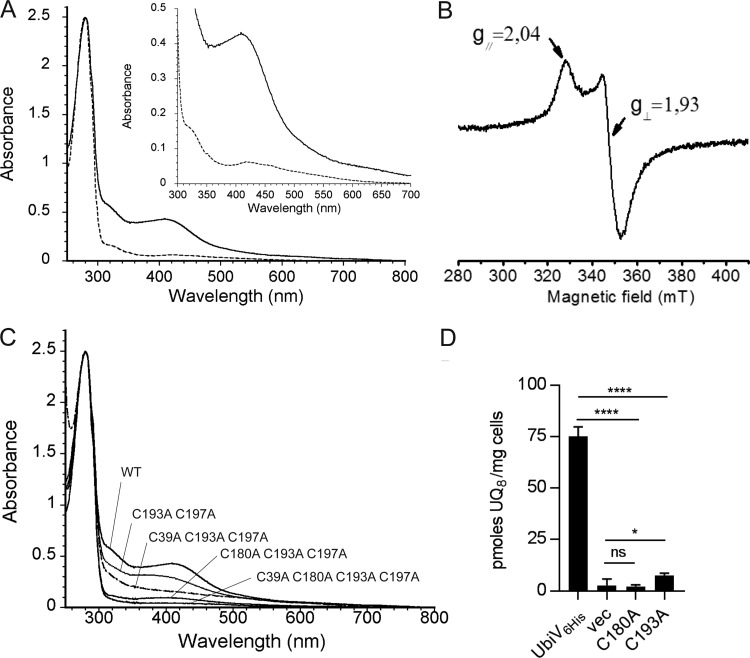
UbiV binds a [4Fe-4S] cluster. (A) UV-visible absorption spectra of as-purified UbiV (dotted line, 47 μM) and reconstituted holo-UbiV (solid line, 41 μM). The inset is an enlargement of the 300- to 700-nm region. The molar extinction coefficient, ε_410nm_, was determined to be 10.8 ± 0.4 mM^−1 ^cm^−1^ for holo-UbiV. (B) X-band EPR spectrum of 785 μM dithionite-reduced holo-UbiV. Recording conditions were the following: temperature, 10K; microwave power, 10 mW; modulation amplitude, 0.6 mT. (C) Comparative UV-visible absorption spectra of WT and different Cys-to-Ala mutants of UbiV after [Fe-S] cluster reconstitution, with the following concentrations: 41 μM WT, 44 μM C193A C197A, 46 μM C39A C193A C197A, 47 μM C180A C193A C197A, and 54 μM C39A C180A C193A C197A. (A to C) Proteins were in 50 mM Tris-HCl, pH 8.5, 25 mM NaCl, 15% glycerol, 1 mM DTT. (D) UQ_8_ quantification of Δ*ubiV* cells transformed with pBAD-UbiV_6His_, pBAD-UbiV_6His_ C180A, pBAD-UbiV_6His_ C193A, or empty pBAD and grown overnight in anaerobic SMGN plus 0.02% arabinose. Values are means ± SD (*n* = 4 to 5). *, *P* < 0.05; ****, *P* < 0.0001; both by unpaired Student's *t* test.

To gain insights into the potential presence of cofactors in UbiU and UbiV, we attempted to characterize them biochemically. UbiU was not soluble but we purified UbiV_6His_, which behaved as a monomer in solution (Fig. S5A and B). UbiV was slightly pink-colored and had a UV-visible (UV-Vis) absorption spectrum with features in the 350- to 550-nm region (Fig. 5A, dotted line), suggesting the presence of iron-sulfur (Fe-S) species (34–36). We indeed detected substoichiometric amounts of iron and sulfur (0.2 Fe and 0.2 S/monomer), indicating oxidative degradation of the [Fe-S] cluster during aerobic purification, as already observed with many other Fe-S proteins (37, 38). Consistent with this hypothesis, anaerobic reconstitution of the [Fe-S] cluster yielded a UbiV protein with 3.9 iron and 3.3 sulfur/monomer (Table 2) and with a UV-Vis spectrum characteristic of a [4Fe-4S]^2+^ cluster (39) (Fig. 5A, solid line) that was affected by exposure to air (Fig. S5C). The electron paramagnetic resonance (EPR) spectrum of the cluster reduced anaerobically displayed features characteristic of a [4Fe-4S]^1+^ cluster in the spin state S = 1/2 (Fig. 5B) (38, 40). Overall, we conclude that, under anaerobic conditions, UbiV is able to bind one air-sensitive, redox-active [4Fe-4S] cluster.

**TABLE 2:** Characterization of UbiV proteins and UbiU-UbiV heterodimeric complexes^*a*^

Proteins	*A*_280_/*A*_410_	Content (nmol/nmol of protein)	
		Iron	Sulfur
UbiV WT	5.8	3.9 ± 0.13	3.3 ± 0.18
UbiV C193A C197A	8.4	2.4 ± 0.02	2.2 ± 0.13
UbiV C39A C193A C197A	15.5	1.9 ± 0.01	1.5 ± 0.4
UbiV C180A C193A C197A	26.7	0.7 ± 0.05	0.6 ± 0.1
UbiV C39A C180A C193A C197A	57.4	0.1 ± 0.04	0
UbiU WT-UbiV WT	4.3	7.9 ± 0.05	7.5 ± 0.06
UbiU C169A C176A-UbiV WT	7.3	4.7 ± 0.0.3	5.8 ± 0.1
UbiU C193A C232A-UbiV WT	8	3.9 ± 0.05	5.8 ± 0.1

a Shown are metal content and UV-Vis properties after attempts of anaerobic reconstitution of their Fe-S centers for both wild-type and mutant proteins.

10.1128/mBio.01319-19.5 FIG S5 (A) Gel filtration chromatogram of aerobically purified UbiV on a Superdex 75 Increase 10/300 GL column. Inset shows Coomassie-staining SDS-PAGE of aerobically purified UbiV. (B) Calibration curve, standard proteins in rhombi, UbiV in circle. (C) UV-visible absorption spectra of 41 μM holo-UbiV under anaerobiosis (solid line) and after 20 min of exposure to air (dotted line). (D) Comparative UV-visible absorption spectra of aerobically purified wild-type UbiV (black) and different Cys-to-Ala mutants of UbiV (C193A C197A in blue, C39A C193A C197A in green, C180A C193A C197A in pink, C39A C180A C193A C197A in brown). Inset shows Coomassie-staining SDS-PAGE of the purifications of UbiV and variants. Lanes: M, molecular mass marker; 1, wild-type UbiV; 2, UbiV C193A C197A; 3, UbiV C39A C193A C197A; 4, UbiV C180A C193A C197A; 5, UbiV C39A C180A C193A C197. (A to D) All proteins were in 50 mM Tris-HCl, pH 8.5, 25 mM NaCl, 15% glycerol, 1 mM DTT. Download FIG S5, TIF file, 2.3 MB. Copyright © 2019 Pelosi et al. 2019 Pelosi et al. This content is distributed under the terms of the Creative Commons Attribution 4.0 International license.

[Fe-S] clusters are typically coordinated by cysteine residues (41, 42), and we obtained evidence that the [4Fe-4S] cluster in UbiV is coordinated by four conserved cysteines arranged in a CX*_n_*CX_12_CX_3_C motif (Fig. S4). Indeed, combinatorial elimination of C39, C180, C193, and C197 in double, triple, and quadruple mutants resulted in proteins incapable of binding [Fe-S] clusters *in vivo*, as shown by the absence of absorption bands in the 350- to 550-nm region of their UV-Vis spectra (Fig. S5D). Furthermore, after anaerobic reconstitution of the cluster, the Fe and S contents and the absorbance at 410 nm were largely decreased in the double and triple mutants and were undetectable in the quadruple mutant (Table 2 and Fig. 5C). Finally, we found that the mutation of C180 or C193 altered the function of UbiV *in vivo* (Fig. 5D), suggesting that the [4Fe-4S] cluster is important for activity.

### UbiU contains a [4Fe-4S] cluster and forms a complex with UbiV.

**FIG 6:**
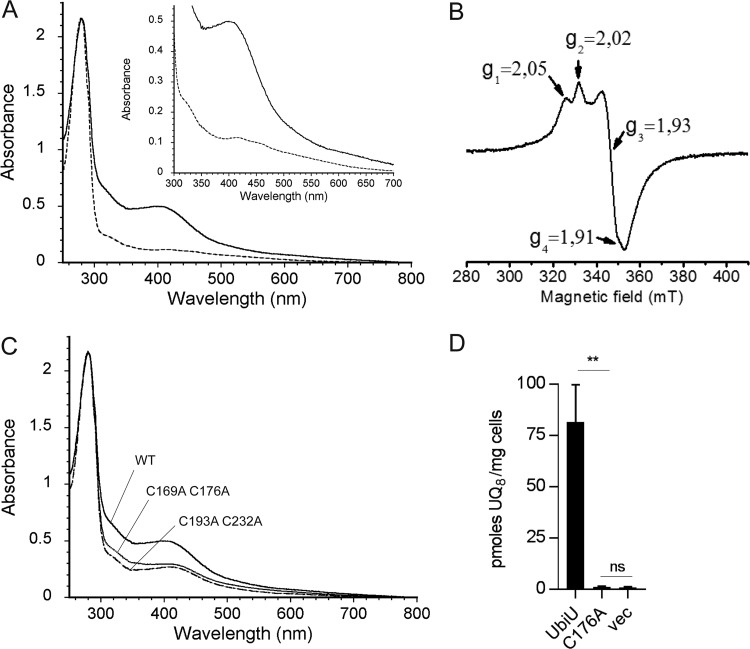
UbiU-V complex binds two [4Fe-4S] clusters. (A) UV-visible absorption spectra of as-purified UbiU-UbiV (dotted line, 17 μM) and reconstituted holo-UbiU-UbiV (solid line, 15.5 μM). The inset shows an enlargement of the 300- to 700-nm region. (B) X-band EPR spectrum of 339 μM dithionite-reduced holo-UbiU-UbiV. Recording conditions were the following: temperature, 10K; microwave power, 2 mW; modulation amplitude, 0.6 mT. (C) Comparative UV-visible absorption spectra of Cys-to-Ala mutants of UbiU in the UbiU-UbiV complex after metal cluster reconstitution with the following concentrations: 15.5 μM WT, 16.0 μM UbiU C169A C176A, and 16.0 μM UbiU C193A C232A. (A to C) Proteins were in 50 mM Tris-HCl, pH 8.5, 150 mM NaCl, 15% glycerol, 1 mM DTT. (D) UQ_8_ quantification of Δ*ubiU* cells transformed with pBAD-UbiU (*n* = 4), pBAD-UbiU C176A (*n* = 2), or pBAD empty vector (*n* = 3) and grown overnight in anaerobic SMGN plus 0.02% arabinose. Values are means ± SD. **, *P* < 0.01; ns, not significant; both by unpaired Student's *t* test.

We succeeded in purifying UbiU after coexpressing it with UbiV_6His_ (Fig. S6A). The two proteins copurified in the form of a heterodimer (Fig. S6A and B) that showed traces of [Fe-S] clusters (Fig. 6A, dotted line), with substoichiometric amounts of iron and sulfide (0.4 Fe and 0.4 S/heterodimer). Reconstitution with iron and sulfide yielded a heterodimer with about 8 iron and 8 sulfur (Table 2) and a UV-visible spectrum characteristic of [4Fe-4S]^2+^ clusters (Fig. 6A, solid line). The EPR spectrum of reduced UbiU-UbiV was also consistent with the presence of 2 different [4Fe-4S] clusters since it showed a composite signal, which reflects the presence of two different S = 1/2 species (Fig. 6B).

10.1128/mBio.01319-19.6 FIG S6 (A) Gel filtration chromatogram of aerobically purified UbiU-UbiV on a Superdex 75 Increase 10/300 GL column. Inset shows Coomassie-staining SDS-PAGE of aerobically purified UbiU-UbiV complex. (B) Calibration curve, standard proteins in rhombi, UbiU-UbiV in circle. (C) Coomassie-staining SDS-PAGE shows the purifications of wild-type UbiU-UbiV and variant proteins. Lanes: M, molecular mass marker; 1, wild-type UbiU-UbiV complex; 2, UbiU C169A C176A-UbiV wild type; 3, UbiU C193A C232A-UbiV wild type. (A to D) All proteins were in buffer of 50 mM Tris-HCl, pH 8.5, 150 mM NaCl, 15% glycerol, 1 mM DTT. Download FIG S6, TIF file, 0.2 MB. Copyright © 2019 Pelosi et al. 2019 Pelosi et al. This content is distributed under the terms of the Creative Commons Attribution 4.0 International license.

Four strictly conserved cysteines are also found in UbiU (Fig. S3), and we hypothesized that they bind the [4Fe-4S] cluster. We eliminated these cysteines in pairs and purified heterodimers composed of WT UbiV_6His_ and mutant UbiU (Fig. S6C). After reconstitution, UbiU C169A C176A-UbiV and UbiU C193A C232A-UbiV had about half the iron as the WT heterodimer (Table 2), and their *A*_280_/*A*_410_ ratios were also diminished about 2-fold (Fig. 6C and Table 2). Altogether, our data clearly demonstrate that each protein of the heterodimeric UbiU-UbiV complex binds one [4Fe-4S] cluster and that the iron-chelating cysteines in UbiU are C169, C176, C193, and C232. Finally, an *in vivo* complementation assay demonstrated that C176 was important for the function of UbiU (Fig. 6D).

### Many U32 proteases display motifs of four conserved cysteines.

**FIG 7:**
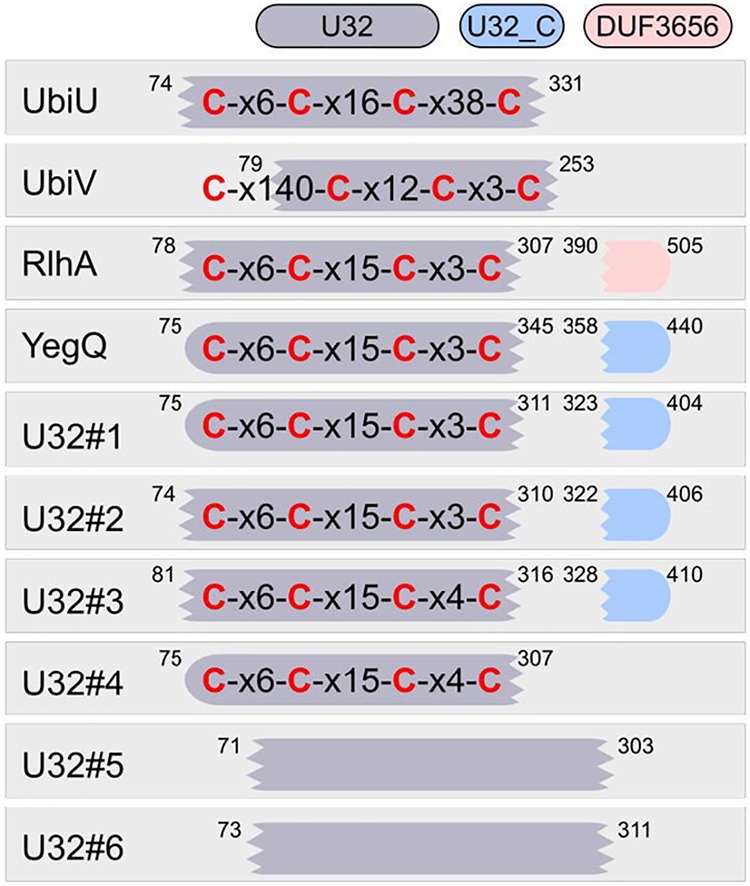
Conserved four-cysteine motifs in the U32 protease family. The conserved 4-cysteine motifs and Pfam domains (colored boxes) found in each U32 protease family are displayed for a set of reference sequences. These motifs were obtained by aligning the sequences listed by Kimura et al. (43). Conserved cysteines are in red, and x6 indicates that 6 residues were found between two conserved cysteines. Positions of the domains are displayed on the outside of the boxes for the reference sequences. Scrambled extremities show interrupted matches for the Pfam domain. No conserved cysteines were found for U32#5 and U32#6 (see the main text). Reference sequences were from E. coli for UbiU, UbiV, YegQ, and RhlA (YHBU_ECOLI, YHBV_ECOLI, YEGQ_ECOLI, and YDCP_ECOLI for RlhA). For the rest of the families, the sequence accession numbers were the following: R7JPV1_9FIRM for U32#1, R6XKQ3_9CLOT for U32#2, S1NZZ5_9ENTE for U32#3, H1YXA1_9EURY for U32#4, H3NJ45_9LACT for U32#5, and D5MIQ1_9BACT for U32#6.

The presence of [Fe-S] clusters in UbiU and UbiV, two U32 protease family members, led us to evaluate the presence of conserved Cys motifs in other U32 proteins. Kimura et al. reported a phylogenetic tree of 3,521 peptidase U32 domains which formed 12 groups, belonging to 10 protein families (43). We extracted and aligned the sequences of the 10 protein families and found highly conserved 4-cysteine clusters (97 to 100% conservation) in eight of them (Fig. 7), suggesting an important functional role for these residues. Only families PepU32#5 and PepU32#6 had no conserved cysteines (PepU32#5) and two mildly (60 to 80%) plus three poorly (40 to 65%) conserved cysteines (PepU32#6) in their sequences. The cysteine motifs for each of the eight families showed a high degree of conservation, and strikingly, most of them could even be aligned with each other, with the CX_6_CX_15_CX_3/4_C patterns appearing recurrently (Fig. 7). Note that UbiV had a slightly distinct motif, with the first cysteine occurring far upstream of the three others and outside of the U32 domain. Overall, our data suggest that most members of the U32 peptidase family contain a [4Fe-4S] cluster coordinated by conserved cysteines, similar to what we demonstrated for UbiU and UbiV.

## DISCUSSION

### The O_2_-independent UQ biosynthesis pathway is widespread in proteobacteria.

The evidence for an O_2_-independent synthesis of UQ by E. coli was reported more than forty years ago (20), yet this pathway remained uncharacterized until now. Circumstantial evidence had been obtained that a few species, limited, to our knowledge, to E. coli (20), Rhodobacter sphaeroides (44), Paracoccus denitrificans (45), and Halorhodospira halophila (46), were able to synthesize UQ under anaerobic conditions, as demonstrated by biochemical measurements of the quinone content of cells grown anaerobically. Here, we demonstrate that the O_2_-dependent and O_2_-independent UQ biosynthesis pathways differ by only three hydroxylation steps (Fig. 1), and we identify three genes, *ubiT*, *-U*, and *-V*, that are essential for the O_2_-independent biosynthesis of UQ in E. coli (Fig. 2). The facts that the UbiT, -U, and -V proteins are widespread in alpha-, beta-, and gammaproteobacterial clades (Fig. 3) and cooccur with UbiA, -E, and -G enzymes (Table S2B) reveal UbiT, -U, and -V as key elements of the broadly distributed, O_2_-independent UQ pathway. Overall, our data support that many proteobacteria have the previously unrecognized capacity to synthesize UQ independently from O_2_.

### Physiological possibilities offered by UQ biosynthesis over the entire O_2_ range.

In our set of reference genomes, only three species (*P. fulvum*, *M. marinus*, and *O. formigenes*) seem to rely exclusively on the O_2_-independent pathway for UQ production (Table S2B). Indeed, the vast majority of proteobacteria with the O_2_-independent UQ biosynthesis pathway also possess the O_2_-dependent hydroxylases of the aerobic pathway (207 out of 210) (Fig. 3B). This result supports that both pathways confer physiological advantages, allowing production of UQ over the entire spectrum of O_2_ levels encountered by facultative aerobes.

So-called microaerobes, able to respire O_2_ under microaerobic conditions, are very abundant in nature (5), and E. coli is known to respire nanomolar O_2_ concentrations (47). To sustain O_2_ respiration in the microaerobic range, these organisms are equipped with high-affinity O_2_ reductases (5, 47, 48). These enzymes reduce efficiently the low levels of environmental O_2_ present at the cell’s membrane, leaving the cytoplasm devoid of any O_2_ (49). Under such conditions, UQ, which is the main electron donor for the high-affinity O_2_ reductases *bd*I and *bd*II of E. coli (9), therefore must be synthesized via the O_2_-independent pathway. Overall, we believe that the O_2_-independent UQ biosynthesis pathway operates not only under anaerobic conditions but also under microaerobic conditions, in which UQ is likely crucial for bacterial physiology.

The O_2_-independent UQ biosynthesis pathway may also confer a significant advantage to facultative bacteria in the case of a rapid transition from an anaerobic to an aerobic environment. Indeed, anaerobic biosynthesis of UQ will result in cellular membranes containing UQ at the time of the transition, allowing an immediate switch to the energetically favorable metabolism of O_2_ respiration. Our identification of the anaerobic UQ pathway provides the unique opportunity to selectively disrupt UQ biosynthesis depending on O_2_ levels and should foster new research on bacterial physiology in the microaerobic range. Indeed, apart from E. coli, which was thoroughly studied over the microaerobic range (4, 49, 50), details on bacterial physiology in microaerobiosis are scarce.

### *ubiT*, *-U*, and -*V* mutants and pathogenicity.

In addition to bioenergetics *per se*, anaerobic and microaerobic respirations are thought to be important for pathogenicity (3, 51). Interestingly, homologs of UbiT, -U, and -V have been linked to pathogenicity in several bacterial models. Indeed, the inactivation of *ubiU-ubiV* homologs in Proteus mirabilis leads to a decreased infection of the urinary tract of mice (52) and to a diminished virulence of Yersinia ruckeri (53), a pathogen that develops in the gut of fish, an environment with a notoriously low O_2_ content. Furthermore, inactivation of PA3911 (54) (*ubiT*) and PA3912-PA3913 (55) (*ubiU-ubiV*) in Pseudomonas aeruginosa abolished nitrate respiration, the main anaerobic metabolism used by the bacterium in the lungs of cystic fibrosis patients (56, 57). Based on our results showing that the deletion of *ubiT*, *ubiU*, or *ubiV* abrogates the O_2_-independent biosynthesis of UQ in E. coli, we suggest that the attenuation of the mutants discussed above results from their UQ deficiency under microaerobic/anaerobic conditions.

### Proposed roles for UbiT, UbiU, and UbiV.

UbiT possesses an SCP2 domain, similar to that of UbiJ, which we recently demonstrated to be an accessory factor that binds the hydrophobic UQ biosynthetic intermediates and structures a multiprotein Ubi complex (16). Since UbiJ functions exclusively in the O_2_-dependent pathway whereas UbiT is important only for the O_2_-independent pathway, we propose that UbiT fulfills, in anaerobiosis, the same functions as UbiJ in aerobiosis. Whether UbiT is part of a complex and is able to bind UQ biosynthetic intermediates will be addressed in future studies. Interestingly, PA3911, the homolog of UbiT in P. aeruginosa, was recently shown to bind phosphatidic acid (54), demonstrating an affinity of UbiT for lipid molecules.

UbiU and UbiV form a tight heterodimer, suggesting that the proteins function together, as further supported by the fact that the *ubiU* and *ubiV* genes cooccur in genomes in 99% of the cases and that they are mostly found next to each other (Fig. 3). We demonstrated that UbiU and UbiV are both required for the O_2_-independent C6-hydroxylation of DMQ, and the accumulation of OPP in Δ*ubiU* or Δ*ubiV* mutants suggests that the two proteins also function in C5-hydroxylation. We want to emphasize our recent demonstration that a single hydroxylase catalyzes all three hydroxylation steps in the O_2_-dependent UQ pathway of Neisseria meningitidis (19). This result showed that three different enzymes are not necessarily required and opens the possibility that UbiU-UbiV in fact catalyze all three hydroxylation reactions of the O_2_-independent UQ biosynthesis pathway. Establishing the hydroxylase activity and the regioselectivity of UbiU-UbiV will require the development of an *in vitro* assay, a challenging task given that the oxygen donor of the reaction is currently unknown and that the substrates are not commercial and are highly hydrophobic. Of note, one of the O_2_-dependent hydroxylases was shown to hydroxylate DMQ_0_, a substrate analog with no polyprenyl side chain (58), suggesting that an *in vitro* assay for UbiU-UbiV can be developed with soluble analogs.

### [Fe-S] clusters in UbiU-UbiV and other members of the U32 peptidase family.

Until now, members of the peptidase family U32 had not been shown to bind [Fe-S] clusters or to contain any of the ∼30 cysteine motifs found in well-characterized iron-sulfur proteins (59). The expression, purification, and spectroscopic characterization of UbiV and of the UbiU-UbiV heterodimeric complex clearly showed that each protein contains one [4Fe-4S] cluster (Fig. 5 and 6). Mutation of the candidate cysteine ligands, arranged in a CX_6_CX_16_CX_38_C motif in UbiU and in a CX*_n_*CX_12_CX_3_C motif in UbiV, disrupted Fe-S binding and abolished *in vivo* complementation, suggesting a crucial function of the [4Fe-4S] clusters in these proteins. The conservation of a CX_6_CX_15_CX_3/4_C motif in other U32 proteases supports that these proteins likely bind [Fe-S] clusters. This hypothesis should guide and stimulate investigations of U32 members, very few of which currently have an established molecular function (http://www.ebi.ac.uk/merops/) (60). Interestingly, RlhA, a member of the U32 protease family involved in the C-hydroxylation of a cytidine on E. coli 23S rRNA, was recently shown to be connected to iron metabolism (43), corroborating our suggestion that RlhA is also an Fe-S protein.

In biological systems, [Fe-S] clusters are mainly known to be involved in electron transfer reactions as well as in substrate binding and activation, in transcription regulation, in iron storage, and as a sulfur donor (41, 61–63). The role of the [Fe-S] clusters in UbiU and UbiV is unknown at this stage. Our current working hypothesis is that they have a role as electron transfer chains between the substrate (the UQ biosynthetic intermediate to be hydroxylated) and an unidentified electron acceptor required for the activation of the substrate. Clearly, the [Fe-S] clusters of UbiU-UbiV are distinct from the molybdenum cofactor present in molybdenum-containing hydroxylases, the only family currently known to catalyze O_2_-independent hydroxylation reactions (64). Together, our results identify UbiU and UbiV as prototypes of a novel class of O_2_-independent hydroxylases and extend the framework of the chemically fascinating O_2_-independent hydroxylation reactions.

## MATERIALS AND METHODS

### Strain construction.

Strains used in this study are listed in Table S3 in the supplemental material. We obtained the collection of E. coli strains containing large and medium deletions from the National BioResource Project, National Institute of Genetics, Japan (http://www.shigen.nig.ac.jp/ecoli/pec/).

10.1128/mBio.01319-19.9 TABLE S3 List of oligonucleotides and strains used in this study. Download Table S3, XLSX file, 0.02 MB. Copyright © 2019 Pelosi et al. 2019 Pelosi et al. This content is distributed under the terms of the Creative Commons Attribution 4.0 International license.

The Δ*ubiA*::*cat*, Δ*ubiD*::*cat*, Δ*ubiT*::*cat*, and Δ*ubiV*::*cat* mutations were constructed in a one-step inactivation of *ubi* genes as described previously (65). A DNA fragment containing the *cat* gene flanked with 5′ and 3′ regions bordering the E. coli *ubi* genes was amplified by PCR using pKD3 as a template and oligonucleotides 5wanner and 3wanner (Table S3). Strain BW25113, carrying the pKD46 plasmid, was transformed by electroporation with the amplified fragment and Cat^r^ colonies were selected. The replacement of chromosomal *ubi* by the *cat* gene was verified by PCR amplification in the Cat^r^ clones. Mutations (including *ubiU*::*kan* from the Keio strain) were introduced into MG1655 strains by P1 *vir* transduction (66), selecting for the appropriate antibiotic resistance. The antibiotic resistance cassettes were eliminated when needed using plasmid pCP20 as described previously (67).

### Plasmid construction.

All plasmids generated in this study were verified by DNA sequencing. The *yhbU*, *yhbT*, and *yhbV* inserts (UniProtKB entries P45527, P64599, and P45475) were obtained by PCR amplification using E. coli MG1655 as the template and the oligonucleotide pairs *yhbU5*-*yhbU3*, *yhbT5*-*yhbT3*, and *yhbV5*-*yhbV3*, respectively (Table S3). *yhb* inserts were EcoRI-SalI digested and inserted into EcoRI-SalI-digested pBAD24 plasmids, yielding the pBAD-*yhbU*, pK-*yhbT*, or pBAD-*yhbV* plasmid, respectively.

To create a plasmid expressing the *ubiV* (*yhbV*) ORF as a C-terminally His-tagged protein, the *ubiV* gene was amplified using pET-22-UbiV-FW (introducing the NdeI site) and pET-22-UbiV-RV (introducing the XhoI site) as primers and pBAD-*yhbV* as the template. The NdeI- and XhoI-digested amplicon was ligated to NdeI- and XhoI-digested pET-22b(+) plasmid to obtain pET-22-UbiV.

The plasmid pETDUET-UbiUV, containing UbiU in multiple cloning site 1 (MCS1) and UbiV in MCS2, was obtained as follows*. ubiU* was amplified from pBAD-*yhbU* using pETDUET-UbiU-FW (introducing the NcoI site) and pETDUET-UbiU-RV (introducing the EcoRI site) as primers. The NcoI- and EcoRI-digested amplicon was ligated to NcoI- and EcoRI-digested pETDUET-1 plasmid to obtain pETDUET-UbiU. The *ubiV* gene was then cloned from pET-22-UbiV into MSC2 of pETDUET-UbiU by PCR amplification with pET-22-UbiV-FW and pETDUET-UbiV-RV (introducing the C-terminal His_6_ tag) as primers. The NdeI- and XhoI-digested amplicon was ligated to NdeI- and XhoI-digested pETDUET-UbiU to obtain pETDUET-UbiUV.

A hexahistidine tag was fused at the N-terminal extremity of UbiV to create pBAD-UbiV_6His_. The *ubiV*_6His_ gene was obtained by PCR amplification (Phusion high-fidelity DNA polymerase) using pBAD-UbiV as a template and 6HisV5 (introducing the NcoI site) and 6HisV3 (introducing the HindIII site and the DNA sequence of the 6× His tag) as primers. The NcoI/HindIII-digested amplicon was cloned into the NcoI/HindII-digested pBAD plasmid.

Variants of UbiV and UbiU were constructed using the Q5 site-directed mutagenesis kit (New England Biolabs) according to the manufacturer’s specifications. The plasmids (pET-22b-UbiV, pETDUET-UbiUV, pBAD-*yhbU*, and pBAD-*yhbV*) were used as templates in conjunction with the appropriate primers for each respective amino acid substitution.

### Culture conditions.

E. coli strains were grown at 37°C in lysogeny broth (LB) medium or in synthetic medium (SM) containing either 0.4% (wt/vol) glycerol, 0.4% (wt/vol) lactate, or 0.2% (wt/vol) glucose as carbon sources. Autoclaved SM medium was supplemented with 0.5% (wt/vol) Casamino Acids and with a 1/100 volume of a filter-sterilized solution of 1 mM CaCl_2_, 200 mM MgCl_2_, 1% (wt/vol) thiamine (68). Ampicillin (50 mg/liter), kanamycin (25 mg/liter), and chloramphenicol (25 mg/liter) were added from stocks (1,000× solution sterilized through 0.22-μm filters and stored at −20°C) when needed. When needed, 0.02% arabinose was added to induce the expression of genes carried on pBAD and pK plasmids. External electron acceptors like KNO_3_ (100 mM) or dimethyl sulfoxide (DMSO; 50 mM) were added to SM for anaerobic cultures. Anaerobic cultures were performed in Hungate tubes containing 12 ml medium deoxygenated by argon (O_2_, <0.1 ppm) bubbling for 25 min before autoclaving (in the case of LB medium, 0.05% antifoam [Sigma] was added). Hungate tubes were inoculated through the septum with 100 μl of overnight precultures taken with disposable syringes and needles from closed Eppendorf tubes filled to the top. Aerobic cultures were performed in Erlenmeyer flasks filled to 1/10 the maximal volume and shaken at 180 rpm.

For the initial screen, we grew ME strains anaerobically in SMGN (SM medium supplemented with glycerol and nitrate). Strains that presented a severe growth defect or a low UQ_8_ content were subsequently grown anaerobically in LB medium.

Cultures were cooled on ice before transferring 5- to 10-ml volumes into 15-ml Falcon tubes for centrifugation at 3,200 × *g* at 4°C for 10 min. Cell pellets were washed in 1 ml ice-cold phosphate-buffered saline and transferred to preweighed 1.5-ml Eppendorf tubes. After centrifugation at 12,000 × *g* at 4°C for 1 min and elimination of supernatant, the cell wet weight was determined (∼10 to 20 mg), and pellets were stored at −20°C prior to quinone extraction. We note that these steps were conducted under normal atmosphere and allowed limited O_2_-dependent UQ biosynthesis in cells grown anaerobically. Thus, modifications (detailed below) were adopted in additional experiments conducted under strict anaerobic conditions.

### Culture under strict anaerobic conditions and cell quenching.

LB medium was supplemented with 100 mg/liter l-cysteine (adjusted to pH 6 with NaOH) and 2.5 mg/liter resazurin. The medium was distributed in Hungate tubes and was deoxygenated by argon (O_2_ < 0.1 ppm) bubbling for 45 min at 60°C. The resazurin was initially purple and then quickly turned to pink, and it eventually became colorless. The Hungate tubes were sealed and autoclaved. Two sequential precultures were performed in order to dilute the UQ present in the initial aerobic inoculum. The first preculture was performed overnight and used Eppendorf tubes filled to the top and inoculated with cells grown aerobically on LB agar. The second preculture was performed for 8 h in Hungate tubes and was used to inoculate Hungate tubes that were subsequently incubated overnight at 37°C. Disposable syringes (1 ml) and needles were flushed 5 times with argon prior to inoculating 50 μl of preculture through the septum of the Hungate tubes. The resazurin remained colorless at all steps of the culture, indicating that the medium in the Hungate tubes was strictly anaerobic. At the end of the culture, the Hungate tubes were cooled on ice for 45 min, and 2 ml medium was sampled through the septum with argon-flushed syringes (2 ml) fitted with needles. The cells were immediately quenched by transfer to −20°C precooled glass tubes containing 6 ml methanol, 0.5-ml glass beads (0.5-mm diameter), and 20 mM KCl. The tubes were homogenized by vortex for 30 s and kept at −20°C prior to quinone extraction. In parallel, we also centrifuged 2 ml of culture from the Hungate tubes in order to determine the weight of the cells and normalized the UQ content of the quenched cells that was subsequently measured.

For the experiments conducted under strict anaerobic conditions (Fig. 2H and I), we used LB medium instead of SMGN, since nitrite, produced during the anaerobic respiration of nitrate in SMGN medium, is able to oxidize resazurin (69).

### Lipid extraction and quinone analysis.

Quinone extraction from cell pellets was performed as previously described (17).

The method for quinone extraction from cells quenched in methanol was slightly adapted from reference 9. Briefly, 4 μl of a 10 μM UQ_10_ solution was added as an internal standard to the cell-methanol mixture. Four ml of petroleum ether (boiling range, 40 to 60°C) was added, the tubes were vortexed for 30 s, and the phases were separated by centrifugation for 1 min at 600 rpm. The upper petroleum ether layer was transferred to a fresh glass tube. Petroleum ether (4 ml) was added to the glass beads and methanol-containing tube, and the extraction was repeated. The petroleum ether layers were combined and dried under nitrogen.

The dried lipid extracts were resuspended in 100 μl ethanol, and a volume corresponding to 1 mg of cell wet weight was analyzed by HPLC-electrochemical detection-mass spectrometry (ECD-MS) with a BetaBasic-18 column at a flow rate of 1 ml/min with mobile phases composed of methanol, ethanol, acetonitrile, and a mix of 90% isopropanol, 10% ammonium acetate (1 M), 0.1% trifluoroacetic acid, mobile phase 1 (50% methanol, 40% ethanol, and 10% mix), and mobile phase 2 (40% acetonitrile, 40% ethanol, and 20% mix). Mobile phase 1 was used in MS detection on an MSQ spectrometer (Thermo Scientific) with electrospray ionization in positive mode (probe temperature, 400°C; cone voltage, 80 V). Single-ion monitoring (SIM) detected the following compounds: OPP (M+NH_4_^+^), *m/z* 656.0 to 656.8, 5 to 10 min, scan time of 0.2 s; DMQ_8_ (M+Na^+^), *m/z* 719 to 720, 6 to 10 min, scan time of 0.2 s; ^13^C_6_-DMQ_8_ (M+Na^+^), *m/z* 725 to 726, 6 to 10 min, scan time of 0.2 s; UQ_8_ (M+NH_4_^+^), *m/z* 744 to 745, 6 to 10 min, scan time of 0.2 s; UQ_8_ (M+Na^+^), *m/z* 749 to 750, 6 to 10 min, scan time of 0.2 s; ^13^C_6_-UQ_8_ (M+Na^+^), *m/z* 755.0 to 756, 6 to 10 min, scan time of 0.2 s; UQ_10_ (M+NH_4_^+^), *m/z* 880.2 to 881.2, 10 to 17 min, scan time of 0.2 s. MS spectra were recorded between *m/z* 600 and 900 with a scan time of 0.3 s. UV detection at 247 nm was used to quantify DMK_8_ and MK_8_. ECD, MS, and UV peak areas were corrected for sample loss during extraction on the basis of the recovery of the UQ_10_ internal standard and were then normalized to cell wet weight. The peak of UQ_8_ obtained with electrochemical detection was quantified with a standard curve of UQ_10_ (17). The absolute quantification of UQ_8_ based on the *m/z* 744.6 signal at 8 min (Fig. 2H and I) was performed with a standard curve of UQ_8_ ranging from 0.5 to 150 pmol UQ_8_ (the detection limit was around 0.1 pmol).

### Anaerobic ^13^C_6_-UQ_8_ biosynthesis activity assay.

Δ*ubiC* Δ*ubiF* cells containing or not containing the additional Δ*ubiU* or Δ*ubiV* deletion were grown overnight in MS medium supplemented with 0.2% glucose. This preculture was used to inoculate, at an optical density at 600 nm (OD_600_) of 0.1, 100 ml of fresh medium supplemented with 10 μM ^13^C_7_-4-HB. The culture was grown at 37°C, 180 rpm, until an OD_600_ of 1, at which point 100 μM 4-HB was added. The cells were pelleted by centrifugation at 3,200 × *g* at 4°C for 10 min and suspended in 100 ml SMGN medium. A 10-ml aliquot was taken for quinone extraction (Fig. 4B to D, aerobiosis), and the rest of the culture was placed at 37°C in an anaerobic bottle with a two-port cap fitted with plastic tubing used to inject argon (O_2_ < 0.1 ppm) throughout the experiment in order to create and maintain anaerobiosis. After 5 min of bubbling, a 10-ml sample was taken corresponding to 0-min anaerobiosis, and then samples were taken every 30 min and analyzed for quinone content.

### Overexpression and purification of proteins. (i) Overexpression and purification of E. coli wild-type UbiV and variants.

The pET-22b(+) plasmid, encoding wild-type UbiV or variants, was cotransformed with pGro7 plasmid (TaKaRa Bio, Inc.) into E. coli BL21(DE3) competent cells. Single colonies obtained from transformation were grown overnight at 37°C in LB medium supplemented with ampicillin (50 μg/ml) and chloramphenicol (12.5 μg/ml). Ten ml of preculture was used to inoculate 1 liter of LB medium with the same antibiotics, and the bacteria were cultured further at 37°C with shaking (200 rpm). At an OD_600_ of 1.2, d-arabinose was added to the cultures at a final concentration of 2 mg/ml. At an OD_600_ of 1.8, the culture was cooled in an ice-water bath, and isopropyl 1-thio-β-d-galactopyranoside (IPTG) was added at a final concentration of 0.1 mM. Cells were then allowed to grow further at 16°C overnight. All subsequent operations were carried out at 4°C. Cells were harvested in an Avanti J-26XP high-performance centrifuge from Beckman Coulter with a JLA-8.1000 rotor at 5,000 × *g* for 10 min. The cell pellets were resuspended in 5 volumes of buffer A (50 mM Tris-HCl, pH 8.5, 150 mM NaCl, 15% [vol/vol] glycerol, 1 mM dithiothreitol [DTT]) containing Complete protease inhibitor cocktail (one tablet per 50 ml) (Roche) and disrupted by sonication (amplitude of 40% for 10 min; Branson digital sonifier). Cell debris was removed by ultracentrifugation in an Optima XPN-80 ultracentrifuge from Beckman Coulter with a 50.2 Ti rotor at 35,000 × *g* for 60 min. The resulting supernatant was loaded onto a HisTrap FF crude column (GE Healthcare) preequilibrated with buffer A. The column was washed with 10 column volumes of buffer B (50 mM Tris-HCl, pH 8.5, 150 mM NaCl, 15% [vol/vol] glycerol, 1 mM DTT, 10 mM imidazole) to remove nonspecifically bound E. coli proteins and then eluted with a linear gradient of 10 column volumes of buffer B containing 500 mM imidazole. Fractions containing WT UbiV or variants were pooled, and phenylmethylsulfonyl fluoride was added at a final concentration of 1 mM. The proteins were then loaded on a HiLoad 16/600 Superdex 75 pg (GE Healthcare) preequilibrated in buffer C (50 mM Tris-HCl, pH 8.5, 25 mM NaCl, 15% [vol/vol] glycerol, 1 mM DTT). The purified proteins were concentrated to 30 to 40 mg/ml using Amicon concentrators (30-kDa cutoff; Millipore), aliquoted, frozen in liquid nitrogen, and stored at −80°C. Overall, a high yield of 150 mg UbiV per liter of culture was obtained.

**(ii) Overexpression and purification of UbiU-UbiV complex and variants.** The overexpression of wild-type UbiU-UbiV complex or variants in E. coli BL21(DE3) competent cells was performed by following the same protocol as that for UbiV. The expression of these proteins was induced by addition of IPTG to a final concentration of 0.05 mM. Wild-type UbiU-UbiV complex or variants were purified with the same procedure as that for UbiV, with the exception that the proteins were loaded on the HiLoad 16/600 Superdex 75 pg with buffer A.

### [Fe-S] cluster reconstitution.

The [Fe-S] cluster(s) reconstitution of holo-UbiV and holo-UbiU/V was conducted under anaerobic conditions in an Mbraun LabStar glove box containing less than 0.5 ppm O_2_. Classically, a solution containing 100 μM as purified UbiV or UbiU/V complex was treated with 5 mM DTT for 15 min at 20°C and then incubated for 1 h with a 5-fold molar excess of both ferrous ammonium sulfate and l-cysteine. The reaction was initiated by the addition of a catalytic amount of the E. coli cysteine desulfurase CsdA (1 to 2% molar equivalent) and monitored by UV-visible absorption spectroscopy. The holo-UbiV or holo-UbiU/V complexes then were loaded onto a Superdex 75 Increase 10/300 GL column (GE Healthcare) preequilibrated with buffer C or A, respectively, to remove all excess iron and l-cysteine. The fractions containing the holoproteins were pooled and concentrated to 20 to 30 mg/ml on a Vivaspin concentrator (30-kDa cutoff).

### Quantification methods.

Protein concentrations were determined using the method of Bradford (Bio-Rad) with bovine serum albumin as the standard. The levels of iron and acid-labile sulfide were determined according to the method of Fish (70) and Beinert (71), respectively.

### UV-Vis spectroscopy.

UV-visible spectra were recorded in a 1-cm-optic-path quartz cuvette under aerobic conditions on a Cary 100 UV-Vis spectrophotometer (Agilent) and under anaerobic conditions in a glove box on a XL-100 Uvikon spectrophotometer equipped with optical fibers.

### EPR spectroscopy.

EPR spectra of frozen solutions were recorded on a Bruker Continuous Wave X-band ELEXSYS E500 spectrometer operating at 9.39 GHz, equipped with an SHQE cavity cooled by a helium flow cryostat (ESR 900; Oxford Instruments) under nonsaturating conditions and using the following parameters: microwave power in the range of 2 to 10 mW and modulation of the magnetic field at 100 kHz, with a modulation amplitude of 0.6 mT. Holo-UbiV or holo-UbiU-UbiV complex was treated with a 10-fold molar excess of dithionite to reduce the [Fe-S] cluster. Each solution was introduced into EPR quartz tubes in a glove box and frozen with liquid nitrogen before the EPR measurements.

### Genome data sets.

The protein sequences from 5,750 complete genomes (extended data set) were downloaded from the NCBI RefSeq database (bacteria and archaea; last accessed in November 2016) (Table S2A). A representative set of complete genomes from a monophyletic group of bacteria that potentially harbor the ubiquinone biosynthesis pathway was also created: “reference” and “representative” genomes were downloaded from the NCBI RefSeq database for 204 *Alphaproteobacteria*, 103 *Betaproteobacteria*, and 303 *Gammaproteobacteria* (last accessed in November 2018). In addition to these 610 genomes, the genome of Phaeospirillum fulvum (99.5% estimated completeness according to CheckM; http://gtdb.ecogenomic.org/genomes?gid=GCF_900108475.1) was included (Table S2B).

### HMM protein profile creation.

An initial set of protein sequences (so-called curated set) was retrieved from genomes manually and from a publication (72) to cover the diversity of UQ-producing organisms. The curated set included 48 pairs of YhbU and YhbV from 10 alpha-, 19 beta-, and 19 gammaproteobacteria, 17 sequences for YhbT, and 64, 181, 69, and 189 sequences for UbiA, MenA, UbiG, and UbiE, respectively (Table S4). For each gene family these sequences then were aligned with Mafft (v7.313, linsi) (73), and each alignment was trimmed at its N-terminal and C-terminal extremities based on the filtering results of BMGE (BLOSUM 30) (74). The core of the alignments were kept as is, and hidden Markov model (HMM) profiles were created directly from the trimmed alignments using the Hmmbuild program (Hmmer suite, version 3.1b2) (75).

10.1128/mBio.01319-19.10 TABLE S4 References of the protein sequences used to build the HMM profiles. Download Table S4, XLSX file, 0.05 MB. Copyright © 2019 Pelosi et al. 2019 Pelosi et al. This content is distributed under the terms of the Creative Commons Attribution 4.0 International license.

To ensure a more sensitive search and good delineation between homologs, phylogenetic curation was used, as YhbU and YhbV are known to be part of the larger U32 protease gene family (43). A search using the YhbU and YhbV HMM profiles was performed with Hmmsearch (Hmmer suite) on the extended 5,750-genome data set, and sequences with an i-evalue (independent e-value) lower than 10E−20 and a coverage of the profiles higher than 90% were selected. The 4,212 sequences obtained were dereplicated using Uclust from the Usearch program suite (80% identity level) (76). A phylogenetic tree was built by maximum likelihood with the IQ-Tree program (best evolutionary model) based on the alignment (Mafft linsi, BMGE with BLOSUM30) of the 480 selected sequences, including all curated YhbU and YhbV sequences (73, 74, 77). YhbU and YhbV proteobacterial sequences formed two separate monophyletic groups, giving credit to our curated set (100% and 80% UF-Boot support, respectively). The other sequences that formed a large monophyletic group of bacterial sequences were categorized as U32 proteases (98% UF-Boot support; https://doi.org/10.6084/m9.figshare.7800614.v1). The 98 sequences from this U32 protease group (Table S4) were used to recreate an HMM profile as described above and served as an outgroup for the profile search.

For YhbT, the first profile obtained from the curated set of sequences was used together with the YhbU, YhbV, and U32 protease profiles for a search in the data set of 611 proteobacterial genomes. A second profile was created from YhbT sequences (YhbT2) (Table S4) that were colocalizing with YhbU and YhbV hits (10E−20 i-evalue and 80% profile coverage). The two YhbT profiles matched complementary sets of sequences and therefore were both used for annotating YhbT in genomes.

A similar approach was taken in order to identify the six known aerobic hydroxylases. Fifty-one Coq7, 73 UbiF, 80 UbiH, 58 UbiI, 24 UbiL, and 32 UbiM sequences were extracted manually and from publications (19, 72) (Table S4, version 1) to serve as a reference, annotated set of sequences. Profiles were created as described above. To ensure their specificity, we ran the HMM profiles against our 5,570-genome data set and selected the sequences that had an i-evalue lower than 10E−20 and a coverage of the profiles higher than 90%. We built two phylogenetic trees as described above, one for Coq7 and another one for UbiFHILM, which are known to be part of the large FMO protein family (19). In the latter case, we dereplicated the 1,619 sequences obtained for the FMO protein family before performing the alignment, alignment filtering, and tree reconstruction steps (using Uclust at the 60% identity level). The Coq7 tree obtained showed our reference Coq7 sequences covered the whole diversity of retrieved sequences, suggesting that they all could be bona fide Coq7 (https://doi.org/10.6084/m9.figshare.7800680). The FMO tree showed a monophyletic group containing all reference FMO ubiquinone hydroxylases, forming subgroups for the different homologs (UbiFHILM) in proteobacteria (https://doi.org/10.6084/m9.figshare.7800620). Further, a large set of sequences formed an outgroup consisting of sequences from various clades of bacteria, many being found outside of proteobacteria, robustly separated from the ubiquinone hydroxylases. We split this large clade into four subtrees and extracted the corresponding sequences to obtain four new HMM profiles (as described above) to be used for precise discrimination between ubiquinone hydroxylases and other members of the FMO family (AlloFMO_1 to AlloFMO_4 in Table S4, version 2). FMO ubiquinone hydroxylase subtrees were also used to redesign improved HMM profiles for UbiFHILM (36, 168, 198, 139, and 65 sequences; see Table S4, version 2).

### Evaluation of genomic distributions with HMMER and MacSyFinder.

Two MacSyFinder models were created to (i) search sequences of interest in genomes using Hmmer and (ii) investigate their genetic architecture (78). A first model was created to focus only on YhbTUV-related genes. In this model, the YhbTUV components were defined as mandatory and U32 protease as accessory. A second, more comprehensive model, Ubi+YhbTUV, was designed to list the families corresponding to the 9 profiles obtained (UbiA, MenA, UbiE, UbiG, 2 YhbT, YhbU, YhbV, and U32 proteases). For both models, the two YhbT profiles were set as exchangeable. The parameter inter_gene_max_space was set to 5 in the YhbTUV model and 10 in the Ubi+YhbTUV model. MacSyFinder was set to run HMMER with the options –i-evalue-select 10E−20 and –coverage-profile 0.8. Independently of their genetic context, sequences corresponding to selected HMMER hits were listed for all profiles in all genomes analyzed in order to establish the genomic distribution for each of the protein families of interest. When several profiles matched a sequence, only the best hit (best i-evalue) was considered.

### U32 protease sequence analysis.

We retrieved from the UniProt-KB database 3,460 protein sequences of U32 proteases that were categorized in 12 families by Kimura et al. (43) and created a FASTA file for each of these families. Fifty sequences from different families could not be retrieved, as they had been deleted from the Uniprot-KB database (46 were obsolete) or were not found based on the published accession numbers. As the RlhA1 and RlhA2 families mostly corresponded to two domains from the same protein sequences that had been split, we put whole sequences together into a single FASTA file for sequence analysis of the overall RlhA family. For each of the 10 families, sequences were dereplicated at the 80% identity level with Uclust in order to limit any potential taxonomic sampling bias, and sequences were aligned (Mafft, linsi). The alignments were visualized in Jalview (79) and used to create the logo sequences. Images of alignments were created using the ESPript webserver (http://espript.ibcp.fr/ESPript/ESPript/) (80).
