# Supplementary figures and images for: Ubiquinone Biosynthesis over the Entire O2 Range: Characterization of a Conserved O2-Independent Pathway
Source: mBio. 2019 Jul 9;10(4):e01319-19. doi: 10.1128/mBio.01319-19 (PMC6747719; doi:10.1128/mBio.01319-19)

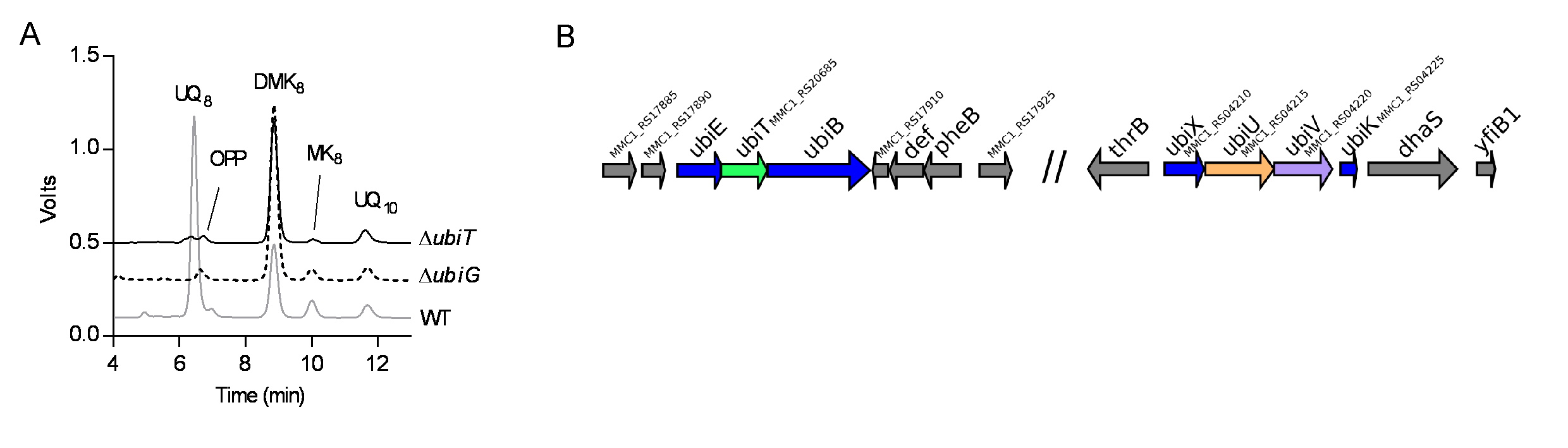

Supplement: FIG S1 [file mBio.01319-19-sf001.jpg]

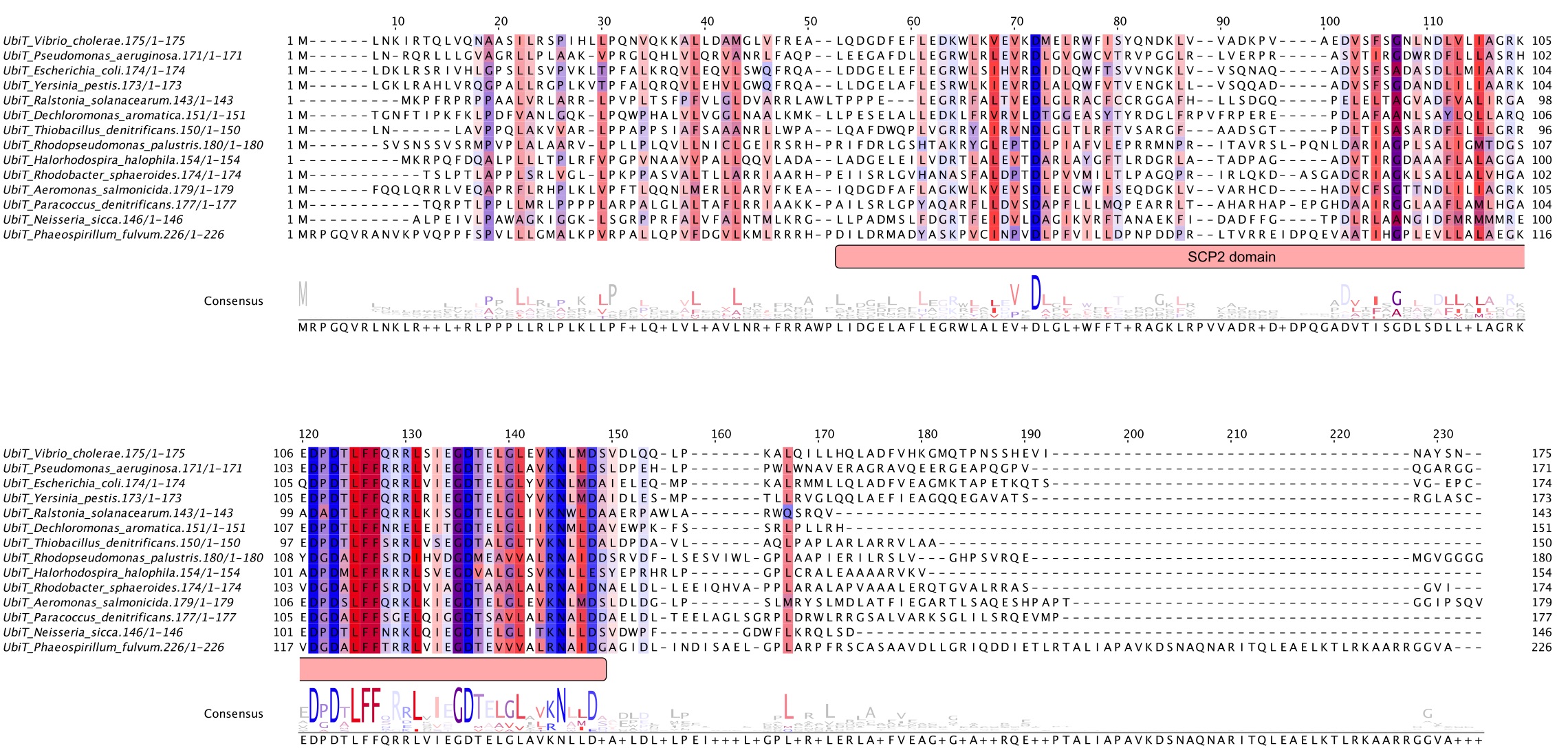

Supplement: FIG S2 [file mBio.01319-19-sf002.jpg]

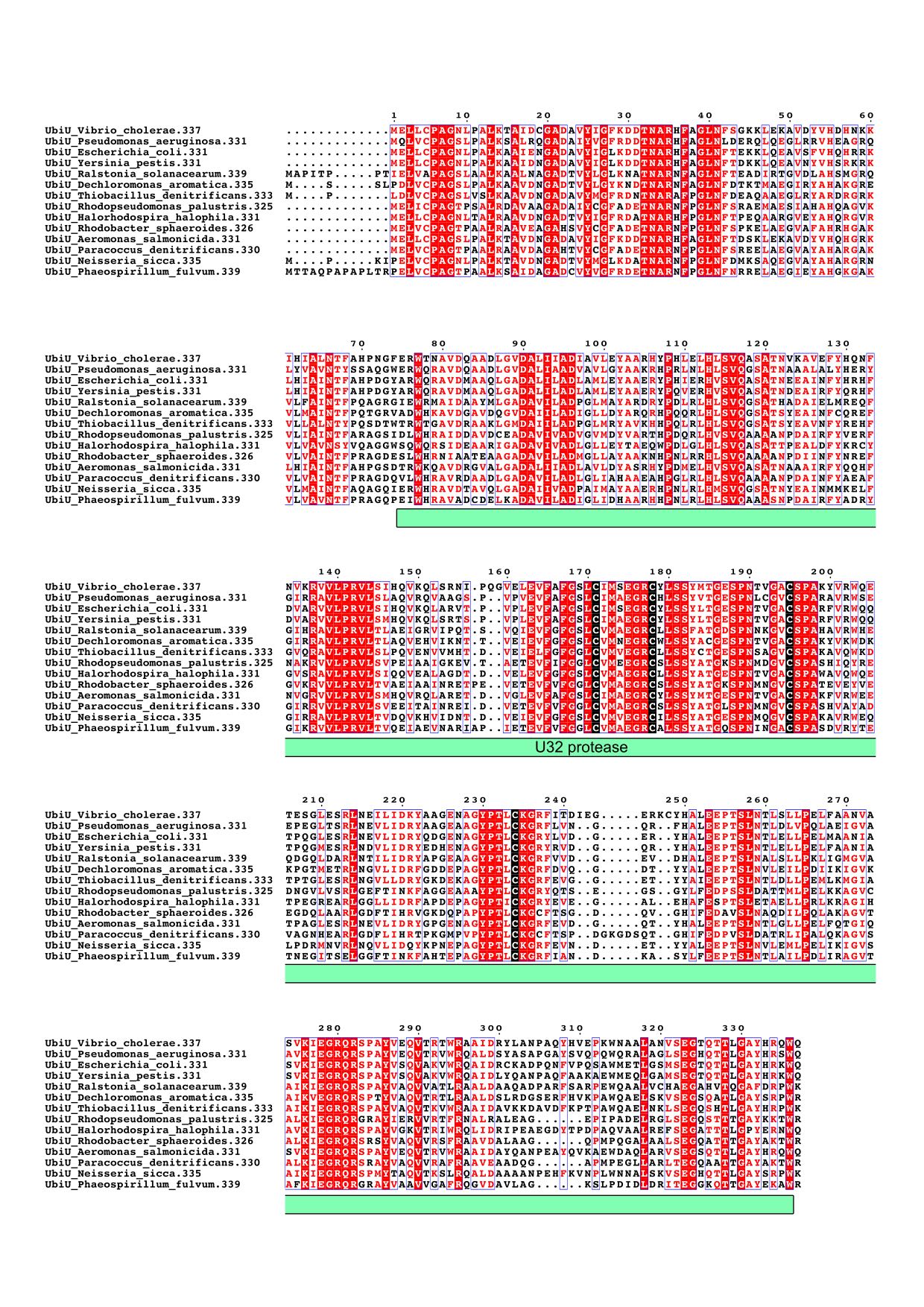

Supplement: FIG S3 [file mBio.01319-19-sf003.jpg]

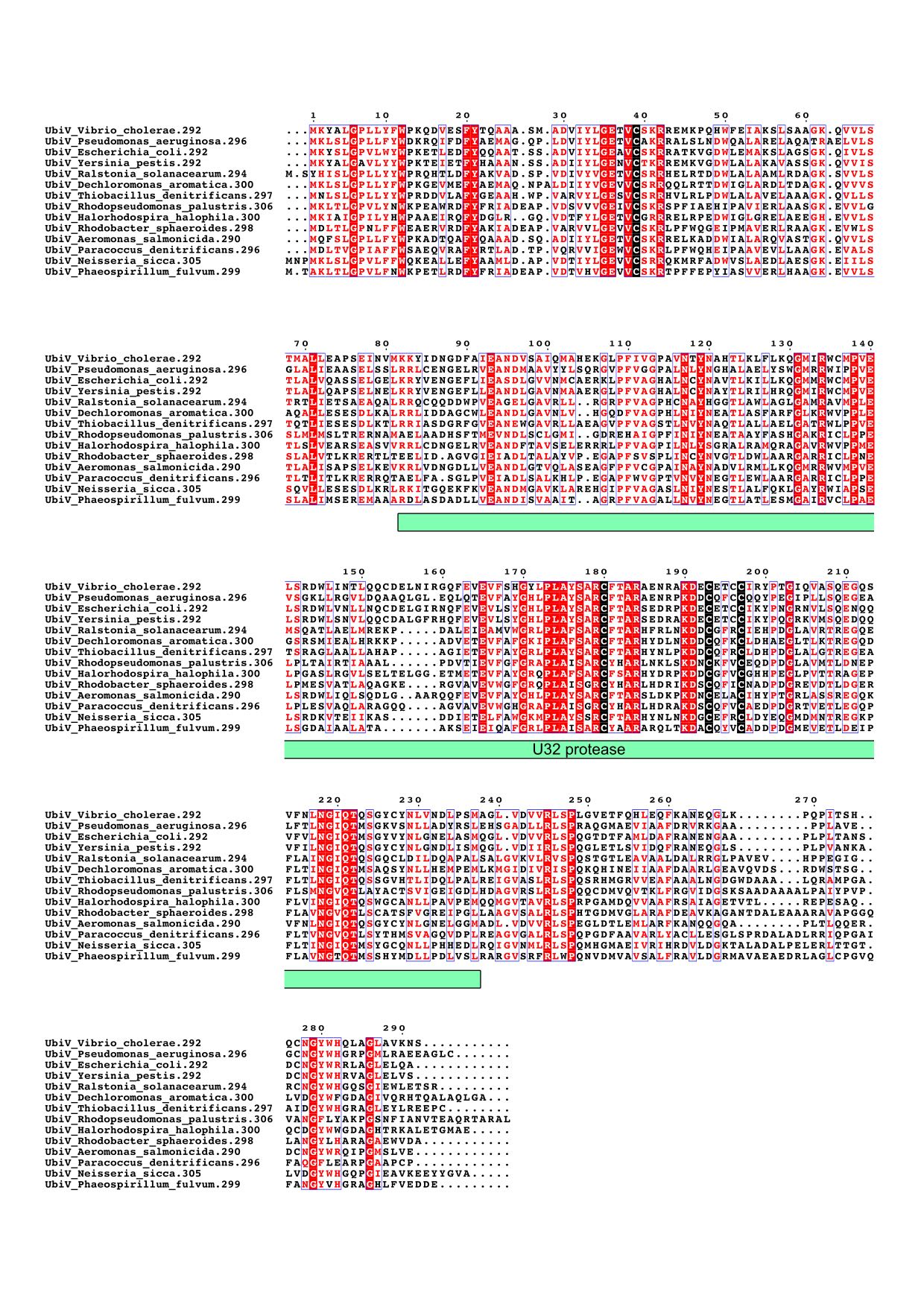

Supplement: FIG S4 [file mBio.01319-19-sf004.jpg]

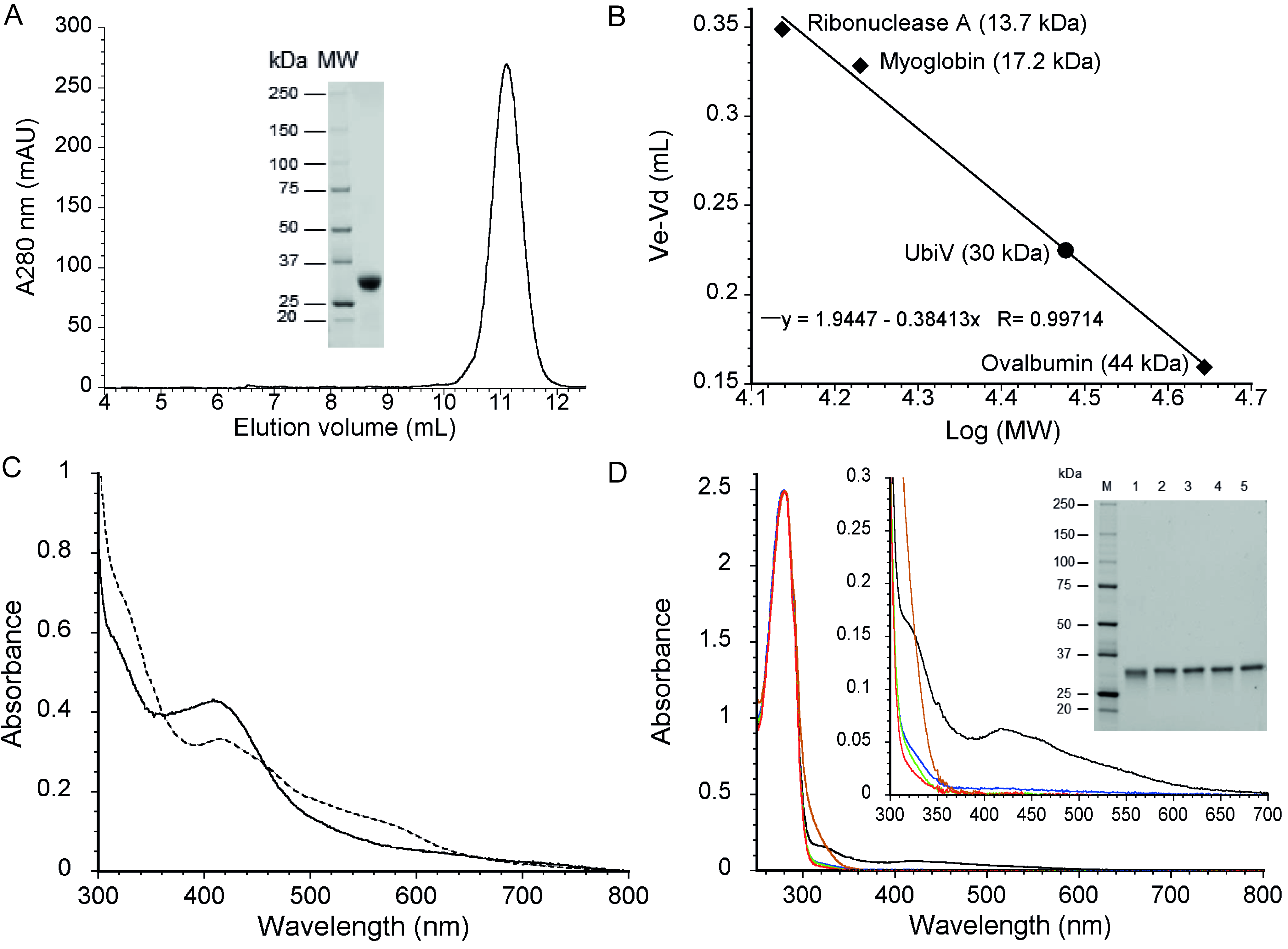

Supplement: FIG S5 [file mBio.01319-19-sf005.tif]

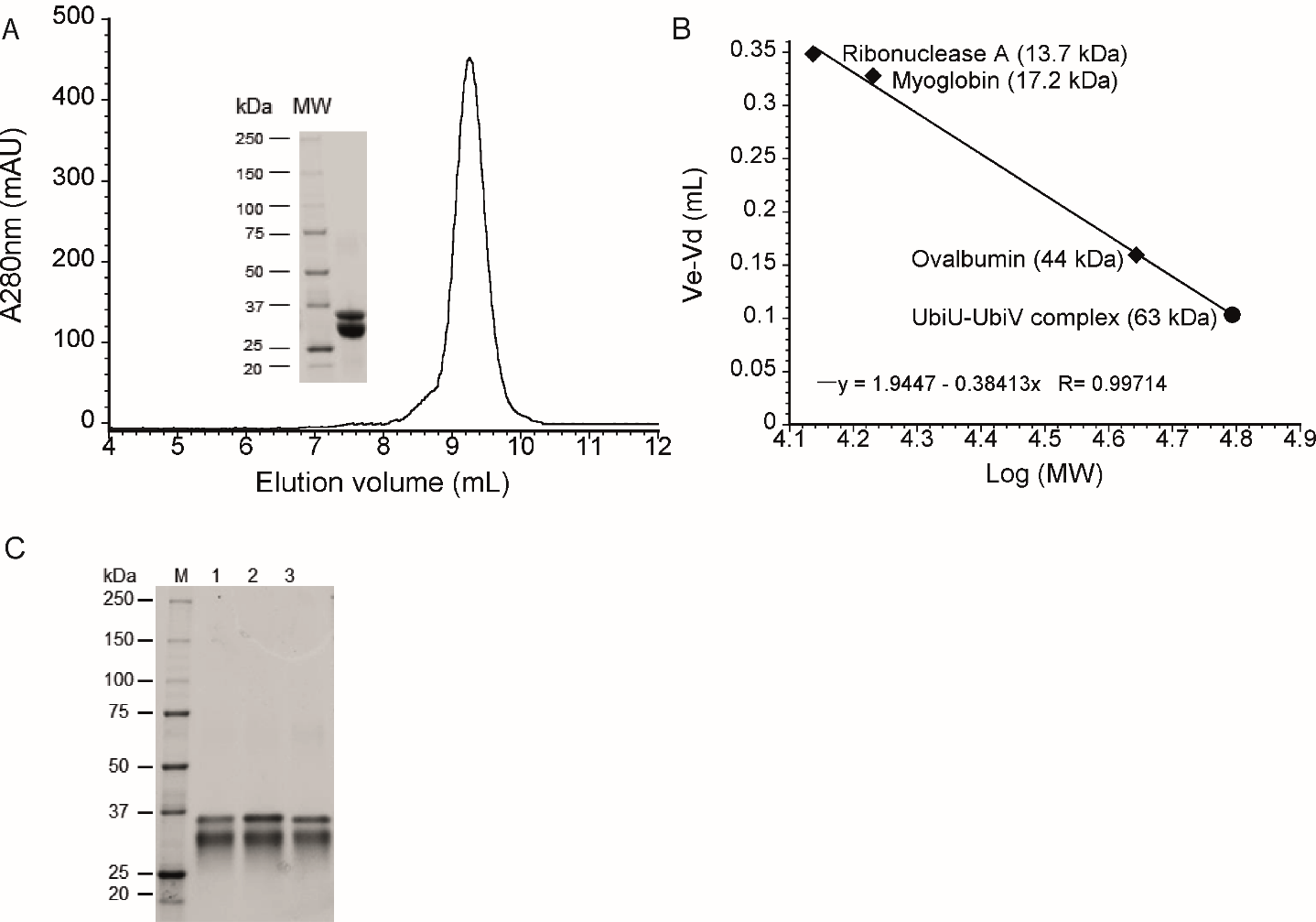

Supplement: FIG S6 [file mBio.01319-19-sf006.tif]
